# Supplementary material for: Host biology, ecology and the environment influence microbial biomass and diversity in 101 marine fish species
Source: Nat Commun. 2022 Nov 17;13:6978. doi: 10.1038/s41467-022-34557-2 (PMC9671965; doi:10.1038/s41467-022-34557-2)
Supplement: Supplementary file 1 — Supplementary Information [file 41467_2022_34557_MOESM1_ESM.pdf]

Supplementary Information
Host biology, ecology, and the environment influence microbial biomass and diversity in 101 marine fish species
Supplementary Note 1. Protocol for performing Katharoseq limit of detection and biomass estimation within Qiime 2.

Microbial Biomass Estimate using 16S amplicon short read data

Author: Jeremiah J Minich [writer], Daniela Perry, Antonio González, Daniel McDonald
Contact: Jeremiah.minich@gmail.com
Date: 7/27/2022
Version: 1.0

Challenge: Microbial biomass (cells per gram or ml) is very difficult to estimate in an environment especially from a host-associated environment

Purpose: Estimate the microbial biomass (bacteria and archaea) from a host associated microbiome

Overview: This SOP will outline both the bench (wetlab) work required for processing samples and controls along with the corresponding computational work to enable the microbial biomass estimation from a sample

Table of contents:
Overview of method	Page 1
Requirements of procedure	Page 2
Part I. Creating the positive controls to be used in the katharoseq method	Pages 3-5
A. microbiology of controls	Page 3
B. making dilutions of the controls	Page 3
C. sample preparation and plating	Page 4
D. DNA extraction	Page 4
E. Library preparation (16S V4 PCR), cleanup, and pooling	Pages 4-5
Part II. Computational processing Katharoseq plugin	Pages 6-14
A. Create biom table to use for processing [Qiita example]	Pages 6-8
B. Determine the read count threshold	Pages 8-12
C. Estimate biomass	Pages 12-14

Additional references:
https://github.com/biocore/q2-katharoseq/blob/master/README.md

1. Minich, J.J., Zhu, Q., Janssen, S., Hendrickson, R., Amir, A., Vetter, R., Hyde, J., Doty, M.M., Stillwell, K., Benardini, J. and Kim, J.H., 2018. KatharoSeq enables high-throughput microbiome analysis from low-biomass samples. *MSystems*, *3*(3), pp.e00218-17.
2. https://www.biorxiv.org/content/10.1101/2022.03.07.483203v1

Requirements:
Consumables and Reagents:

1) Positive controls:
   One or more bacterial isolates with known cell concentrations. We recommend having at least 1 gram + and 1 gram -. For the FMP manuscript we use a *Bacillus subtilis* and *Paracoccus spp*.

   Cell concentrations can be calculated using a variety of methods including standard microbiology plate counts or FACS. If using the plate count method, ensure that counts are determined during the log growth phase so that the actual 'DNA' quants are not underestimated due to dead bacteria.

   One can also buy these from ATCC:
   Paracoccus denitrificans (ATCC 17741) BSL 1
   Bacillus subtilis (ATCC
2) LB agar liquid
3) LB agar plates
4) Micropipettes P1000, P100, P10
5) Filtered tips: 1000, 100, 10
6) Multichannel pipettes or robots
7) DNA extraction kit (validated using hybrid version of Qiagen MagAttract PowerMicrobiome DNA/RNA KF kit (Qiagen Cat# 27600-4-KF).

   Although not tested, protocol should also work with MagMax Microbiome Ultra Nucleic Acid Isolation kit with bead tubes (Thermofisher Cat#A42358)
8) Earthmicrobiome Project 16S primers
9) Earthmicrobiome Project PCR mastermix
10) Molecular grade water
11) PCR cleanup kit: MinElute (Cat# 28004) or QIAquick PCR purification kit (Cat# 28104)
12) Mock communities: Zymo mock (Cat# D6300), ATCC (Cat # MSA-2003)

Equipment

1) Clean workspace such as a PCR setup hood equipped with HEPA filter and UV sterilization
2) Kingfisher robots
3) Thermocyclers (96 well or 384 well)
4) Analytical balance

Part I. Create the positive controls (microbial isolates) to be used in katharoseq

Goal: Prepare and process the controls along with samples through DNA extraction and library prep (16S V4 PCR) using EMP methods. Pool for sequencing.

A. Microbiology of the controls

Prior to processing samples, one needs to 'make' or prepare the controls used in the experiment. Technically one can use any sort of bacteria. We used 1 gram + and 1 gram -.

1. Briefly, a single colony from each bacteria species is inoculated in liquid culture and allowed to be grown up overnight for approximately 12 hours.
2. After 12 hours, take the culture out and quickly dispense ~300 ul into ~25-50 2 ml tubes (on dry ice). This process should not take more than ~10 minutes.
3. Control 1x aliquots should then be stored in the -80 (or -20) until further use.
4. After making aliquots, one should then proceed with serial dilutions: Using sterile technique, make ~8 10 fold serial dilutions into regular sterile media. These dilutions can be made into 2 ml tubes.
5. Briefly mix by swirling around or gentle bump vortexing.
6. For each dilution, pipet 100 ul of the titration onto an agar plate with corresponding media and ensure the culture is evenly distributed. Do this in triplicate at each dilution for a total of ~24 agar plates.
7. Allow agar plates to incubate for at least 12 hours up to 24-36 hours (depending on the microbe used)
8. Determine microbial counts and calculate the original concentration. Label those original stock tubes with the known concentration of that bacteria.

B. Create dilutions of controls:

1. Clean your workspace (such as a clean PCR type hood) by spraying bleach onto all surfaces including pipettes. Allow it to sit for 10 minutes and then wipe down. Spray RNase/DNase onto everything and then wipe down. Lastly, spray everything with 70-95% ethanol to remove residual bleach etc
2. Remove stocks of controls and make ~8 10-fold serial dilutions using molecular grade water.
3. Add 100 ul of each control into a 2 ml bead tube which will later be used for DNA extraction. If desired, you may store this tube in the freezer until further processing but it is recommended to proceed to DNA extraction right away.
4. Include a total of 32 positive controls per experiment. If processing 96 samples at a time, include at least 1 round of positive controls per processing experiment. If possible, always include more positive samples.

C. Sample preparation and plating

For DNA extraction, it is highly recommended to perform the lysis step in a 2 ml bead tube. First, this reduces the amount of well to well contamination [PMID: 31239396] that can occur between samples. Second, its much more practical for weighing the samples. That is, its easier to weigh out tubes before and after adding the sample rather than a whole plate.

1. Determine the total number of samples you wish to extract and then weigh out the corresponding number of 2 ml bead beating tubes using an analytical balance. Record masses in a notebook.
2. In a sterile/DNA free hood, add ~50-200 mg of sample directly to each bead tube.
3. Reweigh each tube using the analytical balance and record masses
4. To determine the mass for each sample subtract to final tube mass from the initial tube mass and record this information in the metadata column:

D. DNA extraction

Follow the general instructions for the DNA extraction kit. For EMP specific instructions, an initial 65 C 10 minute incubation step is performed during the lysis step. For low biomass sample success, it is critical to use an extraction method which utilizes magnetic beads for the cleanup step rather than columns. This protocol was only validated using the PowerMag protocol on the KingFisher robot. While its possible it may work with a column based cleanup, previous work has shown that low biomass samples often do not work well with column methods so the overall limit of detection will suffer. If possible, use the KingFisher Flex robot and be consistent in your elution volume (e.g. 60 ul). Record the elution volume in the metadata column:.

E. PCR and cleanup

1. Follow the EMP 16S instructions outlined elsewhere

2. Briefly, use the same volume of DNA for all PCR reactions. We would recommend using the maximum volume possible (2-5 ul per reaction).
3. You may do a lower overall reaction volume if desired (e.g. 10 ul total volume including 2 ul DNA). [PMID: 30417111]. If you are concerned about low biomass samples, its better to do a higher total PCR reaction volume (25 ul) and use more DNA (5 ul). PMID: 29577086
4. Once PCR finishes, pool the same volume of all samples (e.g. 2 ul) into a single 2 ml tube.
5. Process ~1-2 ml of the volume through a PCR cleanup kit. You may also process the entire volume if you wish.
6. Verify that no primer dimers exist in your pool by running on a gel or through an electronic gel like the TapeStation. If primers still exist, process the pool through a 1 X ampure cleanup
7. Submit final pool for sequencing (e.g. UCSD IGM) and indicate that you are using the EMP 16S protocol. If sequencing on your own instrument, be sure to see the earthmicrobiome.org protocols for the correct custom primers used during sequencing

Part II. Perform the computational analysis to (determine the read control threshold and estimate biomass)

A. Create a collapsed (level-6 = genera) biom table with annotations

You will need to process your raw reads and mapping file through a standard workflow. We recommend using either Qiime2 or Qiita (which wraps Qiime2). Below is a brief example as to how to do this in Qiita. Annotations can be done either with Greengenes or Silva database. Finally, one must collapse this to the genus level. The annotated biom table will be used as input into the Qiime 2 plugin.

1. Process samples in Qiita
2. Create an analysis in Qiita
3. Ensure only samples processed through the methods in Part I are included:

Filter samples to only retain samples from your study of interest
“click” the first triangle on panel (A). “click” process. (B) choose <filter samples…>. (C) type in SQL – in this case we are keeping all samples which have ‘FMP101’ as a string within the “title” metadata column.

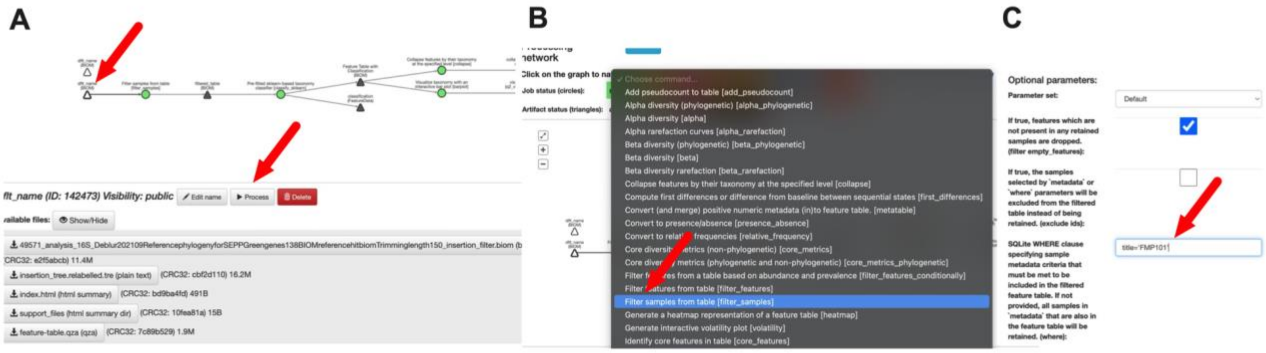

4. Perform the annotation on the biom table

(A) “click” the second triangle and then process. (B) Choose “pre-fitted sklearn …”. (C) Keep everything default, scroll to the bottom and choose either Greengenes or silva. I generally use silva because of working with marine samples.

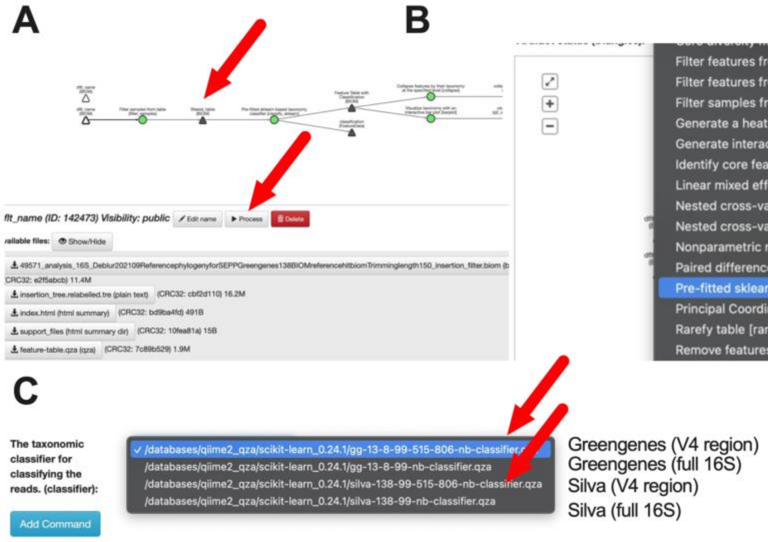

5. Collapse the feature table (biom table) to genus level or level 6 "click" the new triangle and then process.

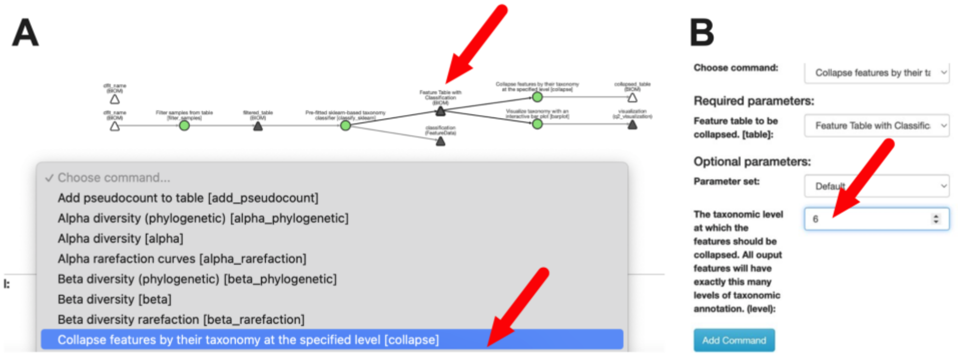

6. Download the biom table and use as input to Qiime 2 (Part IIa and II b). "click" the triangle (collapsed_table), scroll down and download either the feature-table.biom or the collapsed_table.qza (for use in Qiime2)

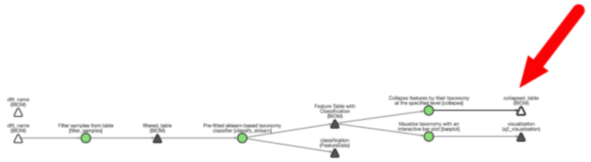

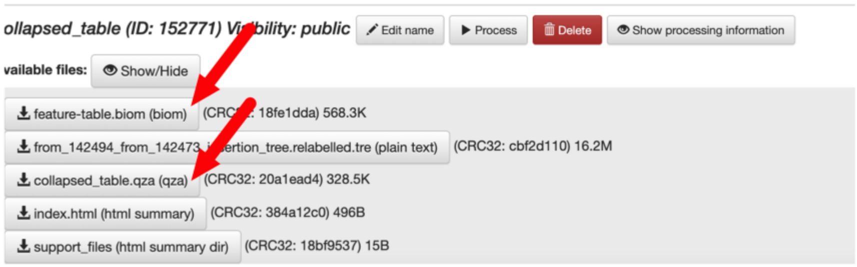

7.Proceed to <**II. determine read control threshold**> and <**III. estimate biomass**> in the Qiime2 environment using the **katharoseq** plugin.

B. Determine the read count threshold using Qiime2 plugin katharoseq

Goal: determine the read count threshold which is the minimum number of reads at which a sample must have to be retained in the analysis

Prior to starting this analysis, ensure that the mapping file includes the following column names. Note, most of these column names can be changed to something else and used accordingly during the Qiime2 commands, but we provide these names as a logical guide.

1. Ensure the "Mapping File" or "metadata" fields contain the following:

MetaData Columns:
control, control_rct, control_cell_into_extraction, control_type, pcr_template_vol, dna_extract_vol, extraction_mass_g, positive_control, total_reads

control:
Note, only ~24-32 samples of 3 (DNA extraction positive control) is required for this protocol but the other types are recommended
1=negative DNA extraction control blank
2=negative DNA extraction control whereby an object (e.g. blank swab) or solution (water) is used as input
3= positive DNA extraction control (a known single species or combination of bacteria species of known concentration)

4=negative control PCR/library prep
5=positive control PCR/library prep (isolated DNA 'some known amount/mass' of a bacteria used as a positive control only during the PCR or library prep stage)

Control_rct: for thte positive controls which you want to use for the read count threshold and biomass estimates, indicate with 'control'
Control = DNA extract positive control samples which you want to use for the calculations

<u>control_cell_into_extraction</u>: Estimate or known total amount of bacteria (control) cells used in the positive control DNA extraction normalized by the final elution volume. This value should be a concentration of cells per ul (eluted DNA). Thus, if you put a total of 1 M cells into the DNA extraction and then eluted at a final volume of 100 ul, you should write the value 10,000 for that sample.

<u>control_type</u>= only refers to the DNA extraction positive controls: either zymo if using zymo mock or KL_mock if using the KL_mock (Bacillus and Paracoccus strains)

<u>pcr_template_vol</u>: a numeric value of the total amount of DNA using as template in the PCR reaction (typically is 1-2 ul but for low biomass we recommend up to 5 ul)

<u>dna_extract_vol</u>: a numeric value of the total elution volume (this is the total final volume of your DNA extraction and is typically between 50 and 100 ul).

<u>extraction_mass_g</u>: this is a numeric value in grams (or

<u>positive_control</u>: Boolean either TRUE if it is the DNA extraction positive control or FALSE (everything else)

Note, the ZymoBIOMICS (Cat# D6300) mock community has an estimated concentration of 1.4 X 10^10 cells / ml

2. Create or obtain a biom table of your samples…this can be done in Qiime2 or Qiita. The original analysis used Qiita, trimmed to 150bp, and ASVs generated using Deblur_v2021.09. Note, this was described in the previous step
3. Annotate the biom table. In the original paper, we used the SILVA 16S database as it generally does better with marine samples than Greengenes but either one should have similar results. Note, this was described in the previous step
4. Collapse the biom table at the genus level or level-6). Note, this was described in the previous step
5. Download Qiime2
   For how to do this, see link below:
   https://docs.qiime2.org/2022.2/install/native/#install-qiime-2-within-a-conda-environment

6. Activate a Qiime2 environment and start the katharoseq command. If the katharoseq plugin is not installed, proceed with the following:

   #within terminal, activate most recent version of Qiime2
   source activate qiime2-2022.2
   git clone https://github.com/biocore/q2-katharoseq
   cd q2-katharoseq
   pip install -e .
   qiime dev refresh-cache

   #to verify you installed katharoseq, correctly type
   Qiime –-help

To ensure you have the most recent version of katharoseq (if you have an old version)
(a) pip uninstall q2_katharoseq

(b)
qiime dev refresh-cache
qiime --help
[shouldn't be there]

(c)
those steps you sent

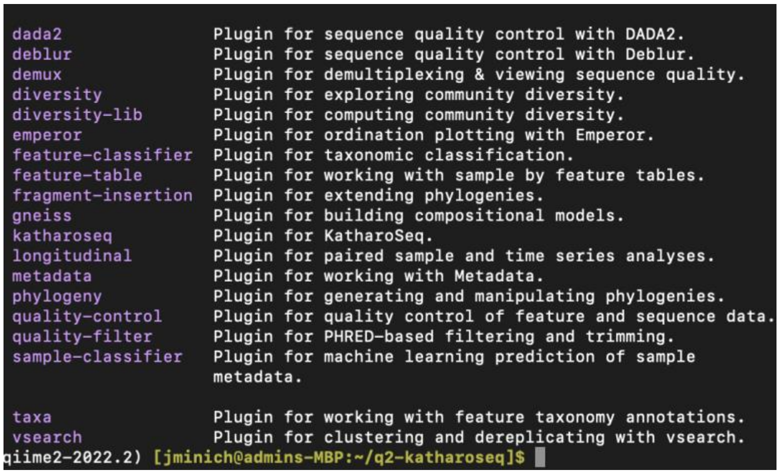

Generate the threshold value (determine the minimum read number at which to retain samples

Details on the method can be found in the readme
https://github.com/biocore/q2-katharoseq/blob/master/README.md

1. Within the Qiime2 environment, proceed with the following:

Read Count Threshold

In order to obtain a read count threshold, computation of a minimum read count threshold can be performed with the `read-count-threshold` plugin action. Test data can be found under the `example` folder.

```
qiime katharoseq read-count-threshold \
    --i-table example/fmp_collapsed_table.qza \
    --m-positive-control-column-file example/fmp_metadata.tsv \
    --m-positive-control-column-column control_rct \
    --m-cell-count-column-file example/fmp_metadata.tsv \
    --m-cell-count-column-column control_cell_into_extraction \
    --p-positive-control-value control \
    --p-control classic \
    --p-threshold 90 \
    --o-visualization result_fmp_example.qzv
```

Overview:
--i refers (generally refers to input files)
--p parameters (you can change)
--m (fields refers to fields 'column names' related to the metadata table)
--o (output)

Details:
--i-table qiime2_plugin_test/152771_collapsed_table.qza \ #this is your collapsed at level-6 genera biom table

--m-positive-control-column-file qiime2_plugin_test/fmp_metadata_mod.tsv \ #actual metadata file

--m-positive-control-column-column control_rct # name of the metadata column indicating the positive control

--m-cell-count-column-file qiime2_plugin_test/fmp_metadata_mod.tsv \ #actual metadata file

--m-cell-count-column-column control_cell_into_extraction \#estimated number of control cells which went into the extraction

--p-positive-control-value control \ #this is the actual text which indicates which samples you wish to use as your positive control. The value is from the column used above in the –m-positive-control-column-column … in this case we indicate our positive controls with the value 'control' to make it clear.

--p-control classic \ #type of control used: ‘zymobiomics, atcc, classic’. ‘classic’ refers to the combination of Bacillus and Paracoccus

--p-threshold 90 \
#value at which the threshold is set. This is the percent (relative abundance) of reads which map back to the known control values. For instance, if you’re sequencing the Bacillus – Paracoccus classic mock community, if you have no contamination then you will have 100% of the reads mapping or being assigned/classified as Bacillus or Paracoccus. As you have lower biomass samples, your opportunity for contamination to show up increases. We set this threshold to 90 to determine the read count of a control sample whereby 90% of the reads are actually from the known or expected microbe. It is possible to modify this number to be lower, but it is up to the discernment of the researcher.

--o-visualization result.qzv

2. To visualize the output, in a web browser: go to view.qiime2.org

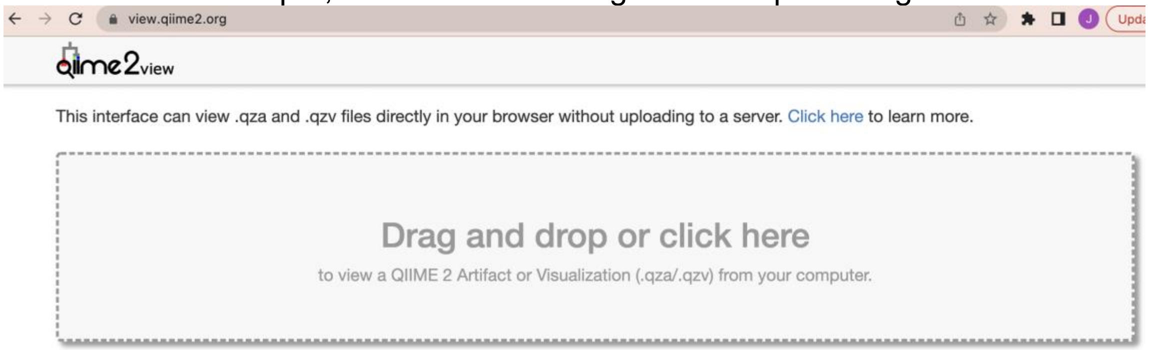

3. Drop in the newly created qzv file
4. Look at the output

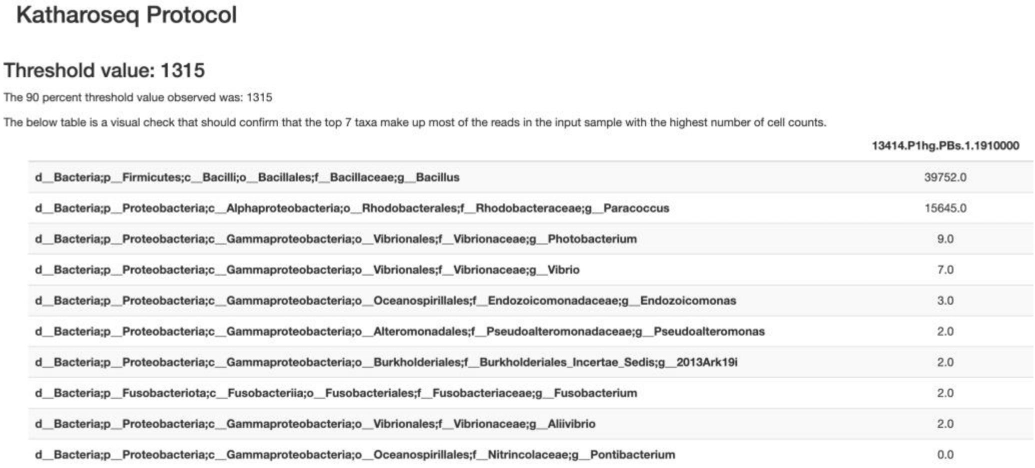

Katharoseq Protocol

Threshold value: 1315

The 90 percent threshold value observed was: 1315

The below table is a visual check that should confirm that the top 7 taxa make up most of the reads in the input sample with the highest number of cell counts.

	13414.P1hg.PBs.1.1910000
d__Bacteria;p__Firmicutes;c__Bacilli;o__Bacillales;f__Bacillaceae;g__Bacillus	39752.0
d__Bacteria;p__Proteobacteria;c__Alphaproteobacteria;o__Rhodobacterales;f__Rhodobacteraceae;g__Paracoccus	15645.0
d__Bacteria;p__Proteobacteria;c__Gammaproteobacteria;o__Vibrionales;f__Vibrionaceae;g__Photobacterium	9.0
d__Bacteria;p__Proteobacteria;c__Gammaproteobacteria;o__Vibrionales;f__Vibrionaceae;g__Vibrio	7.0
d__Bacteria;p__Proteobacteria;c__Gammaproteobacteria;o__Oceanospirillales;f__Endozoicomonadaceae;g__Endozoicomonas	3.0
d__Bacteria;p__Proteobacteria;c__Gammaproteobacteria;o__Alteromonadales;f__Pseudoalteromonadaceae;g__Pseudoalteromonas	2.0
d__Bacteria;p__Proteobacteria;c__Gammaproteobacteria;o__Burkholderiales;f__Burkholderiales_Incertae_Sedis;g__2013Ark19i	2.0
d__Bacteria;p__Fusobacteriota;c__Fusobacteriia;o__Fusobacteriales;f__Fusobacteriaceae;g__Fusobacterium	2.0
d__Bacteria;p__Proteobacteria;c__Gammaproteobacteria;o__Vibrionales;f__Vibrionaceae;g__Aliivibrio	2.0
d__Bacteria;p__Proteobacteria;c__Gammaproteobacteria;o__Oceanospirillales;f__Nitrincolaceae;g__Pontibacterium	0.0

In this case, we determine the read count threshold to be 1315 reads. This differs from the 1150 value used in the fmp manuscript because the example includes an additional sequencing file. The purpose is to demonstrate this as an example.

C. Estimate biomass using Qiime2 plugin katharoseq

Goal: Estimate the microbial biomass per sample (16S copies per g or ml of tissue). Using the output from previous step

1. Run the following command within the Qiime2 environment

Estimating Biomass

Estimate the biomass of samples using KatharoSeq controls. After obtaining a read count threshold using the action above, use the same metadata and collapsed table as input. The `--p-pcr-template-vol` and `--p-dna-template-vol` values are numeric values that should come from your experimental procedures.

```
qiime katharoseq estimating-biomass \
    --i-table example/fmp_collapsed_table.qza \
    --m-control-cell-extraction-file example/fmp_metadata.tsv \
    --m-control-cell-extraction-column control_cell_into_extraction \
    --p-min-total-reads 1315 \
    --p-positive-control-value control \
    --m-positive-control-column-file example/fmp_metadata.tsv \
    --m-positive-control-column-column control_rct \
    --p-pcr-template-vol 5 \
    --p-dna-extract-vol 60 \
    --m-extraction-mass-g-column extraction_mass_g \
    --m-extraction-mass-g-file example/fmp_metadata.tsv \
    --o-estimated-biomass estimated_biomass_fmp_rct
```

--i-table qiime2_plugin_test/152771_collapsed_table.qza \ #this is your collapsed at level-6 genera biom table

--m-control-cell-extraction-file qiime2_plugin_test/fmp_metadata_mod.tsv \ #actual metadata file

--m-control-cell-extraction-column control_cell_into_extraction \ #number of control cells used as input into extraction

--p-min-total-reads 1315 \ #value obtained from previous step ‘read count threshold’

--m-positive-control-column-file qiime2_plugin_test/fmp_metadata_mod.tsv \ #actual metadata file

--m-positive-control-column-column control_rct # name of the metadata column indicating the positive control

--p-pcr-template-vol 5 \ #number of ul of gDNA used as template into the PCR reaction, typically 1-5 ul

--p-dna-extract-vol 60 \ #final elution volume of DNA extraction, typically 50-100 ul

Supplementary Figure 1

--m-extraction-mass-g-column extraction_mass_g \ #actual mass in grams of tissue used in the extraction
--m-extraction-mass-g-file qiime2_plugin_test/fmp_metadata_mod.tsv \ #metadata file
--o-estimated-biomass estimated_biomass_fmp_rct #final output file which contains the estimated number of 16S copies per gram of tissue for each sample

2. Export the qza file to a text file for futher interpretation
https://docs.qiime2.org/2022.2/tutorials/exporting/

```
#to export the qza file and convert to a .csv file for further analysis
qiime tools export \
--input-path estimated_biomass_fmp_rct.qza \
--output-path exported_estimated_biomass_fmp_rct
```

2. Run this command within the Qiime2 environment to visualize results
   This will generate a plot of your positive control values (reads vs. known cells)

Biomass Plot

Finally in order to visualize the results from `estimating-biomass`, run `biomass-plot`.

```
qiime katharoseq biomass-plot \
    --i-table example/fmp_collapsed_table.qza \
    --m-control-cell-extraction-file example/fmp_metadata_mod.tsv \
    --m-control-cell-extraction-column control_cell_into_extraction \
    --p-min-total-reads 1315 \
    --p-positive-control-value control \
    --m-positive-control-column-file example/fmp_metadata_mod.tsv \
    --m-positive-control-column-column control_rct \
    --o-visualization biomass_plot_fmp
```

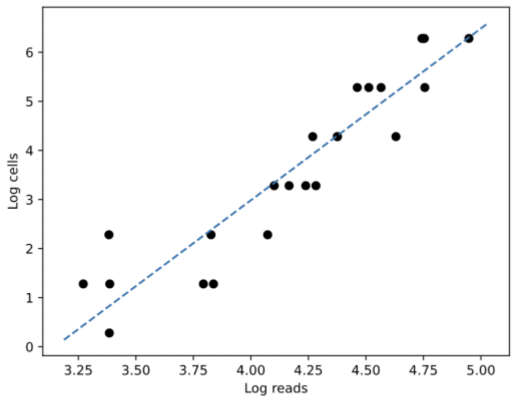

Supplementary Note 1

Supplementary Figure 1

Red=gill only [Atlantic]

Class	Order	Family	Species/FMP number	
Actinopterygii	Albuliformes	Albulidae (bonefishes)	Albula vulpes<br>bonefish<br>FMP 50	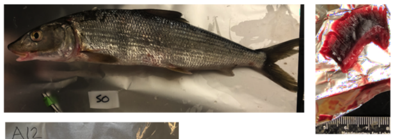
	Anguilliformes	Anguillidae (freshwater eels)	Anguilla rostrata<br>american eel<br>FMP 113 (A12)	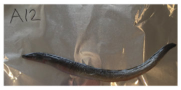
		Muraenidae (moray eels)	Gymnothorax mordax<br>california moray<br>FMP 68	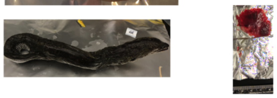
		Nemichthyidae (snipe eels)	Nemichthys scolopaceus<br>snipe eel<br>FMP 78	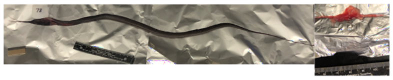
		Nettastomatidae (duckbill eels)	Facciolella equatorialis<br>dog face witch eel<br>FMP 86	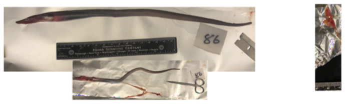
	Argentiniformes	Bathylagidae (deep-sea smelts)	Leuroglossus stilbius<br>CA Smoothtongue<br>FMP 77	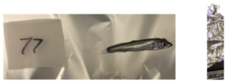
			Lipolagus ochotensis<br>Earned Blacksmelt<br>FMP 70	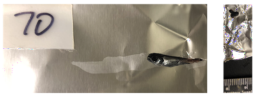
	Atheriniformes	Atherinopsidae (neotropical silversides)	Atherinops affinis<br>topsmelt<br>FMP 99	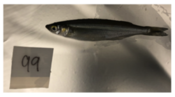
			Atherinopsis californiensis<br>jacksmelt<br>FMP 19	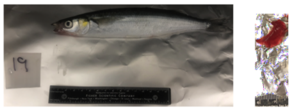
	Aulopiformes	Synodontidae (lizardfishes)	Synodus lucioceps<br>CA lizardfish<br>FMP 51	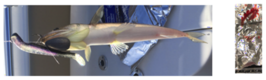
	Batrachoidiformes	Batrachoididae (toadfishes)	Opsanus tau<br>oyster toadfish<br>FMP 112 (A11)	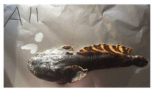
			Porichthys myriaster<br>specklefin midshipman<br>FMP 48	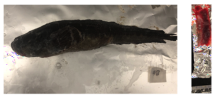
			Porichthys notatus<br>plainfin midshipman<br>FMP 72	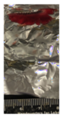
	Beloniformes	Belonidae (needlefishes)	Strongylura marina<br>Atlantic needlefish<br>FMP 111 (A10)	

Class	Order	Family	Species/FMP number<br>Red=gill only [Atlantic]	
Actinopterygii	Beryciformes	Melamphaidae (bigscale fishes/ Ridgeheads	Scopelogadus bispinosus<br>Twospine Bigscale<br>FMP 87	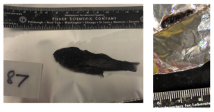
	Blenniformes	Blenniidae (combtooth blennies)	Chasmodes bosquianus<br>Striped Blenny<br>FMP 110 (A9)	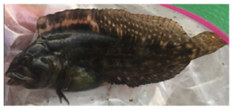
			Hypsoblennius gilberti<br>Rockpool blenny<br>FMP 34	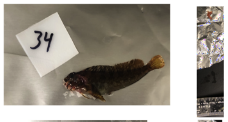
		Clinidae (clinids)	Gibbonsia elegans<br>Spotted Kelpfish<br>FMP 33	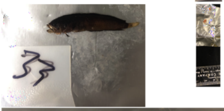
			Heterostichus rostratus<br>Giant Kelpfish<br>FMP 23	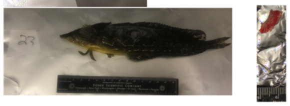
	Carangiformes	Carangidae (jacks and pompanos)	Caranx crysos<br>Blue Runner<br>FMP 114 (A13)	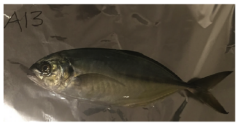
			Seriola dorsalis<br>CA yellowtail<br>FMP 79	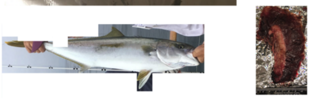
			Trachurus symmetricus<br>Pacific jack mackerel<br>FMP 20	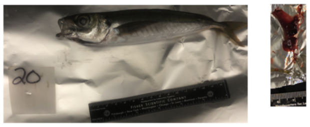
		Coryphaenidae (dolphinfishes)	Coryphaena hippurus<br>Dolphinfish<br>FMP 115 (A14)	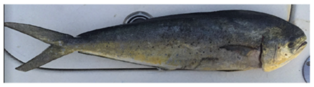
			Coryphaena hippurus<br>Dolphinfish<br>FMP 97	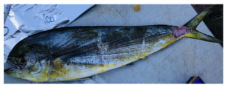
		Sphyraenidae (barracudas)	Sphyraena argentea<br>Pacific 'CA' Barracuda<br>FMP 11	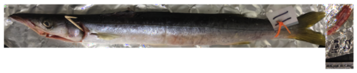
	Centrarchiformes	Girellidae	Girella nigricans<br>Opaleye<br>FMP 22	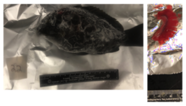
		Kyphosidae (sea chub)	Kyphosus azurea<br>Zebra Perch<br>FMP 21	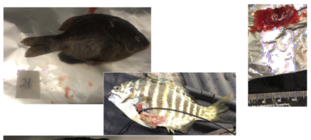
			Medialuna californiensis<br>Halfmoon<br>FMP 54	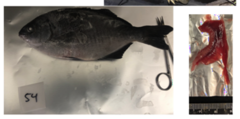

Class	Order	Family	Species/FMP number<br>Red=gill only [Atlantic]	
Actinopterygii	Clupeiformes	Clupeidae<br>(herring, shad, sardine, & menhadens)	Brevoortia tyrannus<br>Atl menhaden<br>FMP 109 (A8)	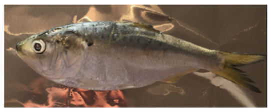
			Sardinops sagax<br>sardine<br>FMP 60	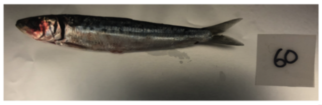 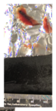
		Engraulidae<br>(anchovies)	Engraulis mordax<br>CA anchovy<br>FMP 40	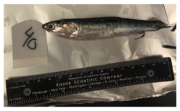 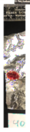
	Cyprinodontiformes	Fundulidae<br>(topminnows and killisfishes)	Fundulus heteroclitus<br>mummichog<br>FMP 108 (A5)	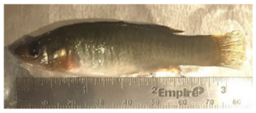
			Fundulus parvipinnis<br>California killifish<br>FMP 96	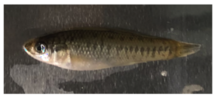
			Lucania parva<br>Rainwater Killifish<br>FMP 116 (A18)	
	Gadiformes	Macrouridae<br>(grenadiers or rattails)	Nezumia stelgidolepis<br>California Grenadier<br>FMP 93	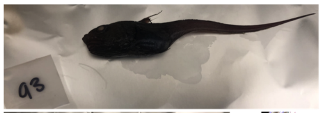 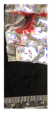
		Merlucciidae<br>(merluccid hakes)	Merluccius productus<br>North Pacific Hake<br>FMP 74	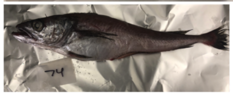 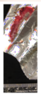
		Moridae<br>(morid cods)	Physiculus rastrelliger<br>Hundred Fathom Mora<br>FMP 64	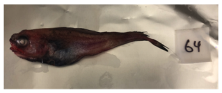 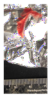
	Gobbiformes	Gobiidae<br>(gobbies)	Clevelandia ios<br>Arrow Goby<br>FMP 95	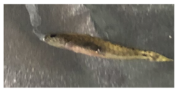
			Gillichthys mirabilis<br>Longjaw Mudsucker<br>FMP 100	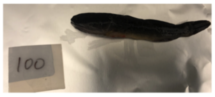
	Labriformes	Labridae<br>(wrasses)	Halichoeres semicinctus<br>Rock Wrasse<br>FMP 8	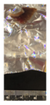
			Oxyjulis californica<br>Senorita<br>FMP 24	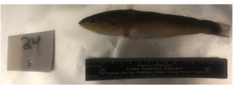 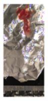
			Semicossyphus pulcher<br>CA Sheephead<br>FMP 18	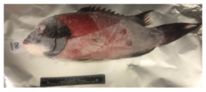 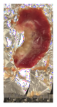

Class	Order	Family	Species/FMP number<br>Red=gill only [Atlantic]	
Actinopterygii	Myctophiformes	Myctophidae<br>(lanternfishes)	Ceratoscopelus townsendi<br>Dogtooth Lampfish<br>FMP 91	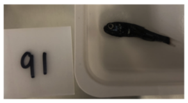 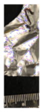
			Nannobrachium ritteri<br>Broadfin lampfish<br>FMP 88	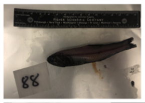 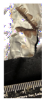
			Triphoturus mexicanus<br>Mexican Lampfish<br>FMP 61	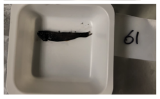 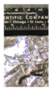
	Pempheriformes	Polyprionidae<br>(wreckfishes)	Stereolepis gigas<br>Giant Seabass<br>FMP 92	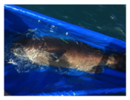 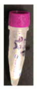
	Perciformes	Agonidae<br>(poachers)	Xeneretmus latifrons<br>Blacktip Poacher<br>FMP 69	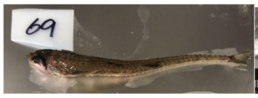 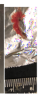
		Cottidae<br>(sculpins)	Leptocottus armatus<br>Pacific Staghorn Sculpin<br>FMP 16	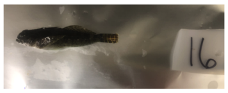 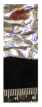
		Embiotocidae<br>(surfperches)	Embiotoca jacksoni<br>Black Perch<br>FMP 9	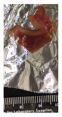
			Phanerodon furcatus<br>White Seaperch<br>FMP 7	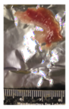
			Brachyistius frenatus<br>Kelp Perch<br>FMP 53	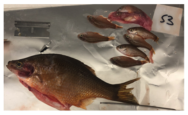 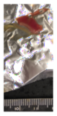
			Rhacochilus toxotes<br>Rubberlip Seaperch<br>FMP 52	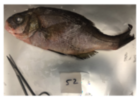 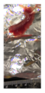
		Haemulidae<br>(grunts)	Anisotremus davidsonii<br>Xantic Sargo<br>FMP 10	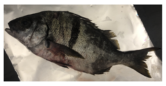 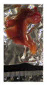
			Xenistius californiensis<br>CA Salema<br>FMP 45	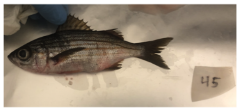 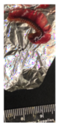
		Malacanthidae<br>(tilefishes)	Caulolatilus princeps<br>Ocean Whitefish<br>FMP 2	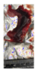

Class	Order	Family	Species/FMP number<br>Red=gill only [Atlantic]	
Actinopterygii	Perciformes	Moronidae (temperate basses)	Morone saxatilis<br>Striped Bass<br>FMP 120 (A23)	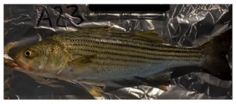
		Pomacentridae (damselfishes)	Chromis punctipinnis<br>Blacksmith<br>FMP 36	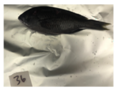 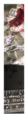
		Sciaenidae (drums & croakers)	Bairdiella chrysoura<br>Silver Perch<br>FMP 107 (A4)	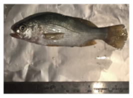
			Cheilotrema saturnum<br>Black Croaker<br>FMP 102	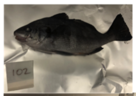
			Cynoscion parvipinnis<br>Shortfin Weakfish<br>FMP 49	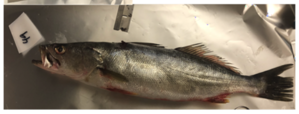 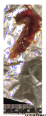
			Menticirrhus undulatus<br>CA Kingcroaker<br>FMP 30	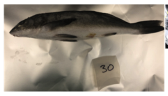 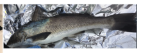 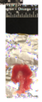
			Roncador stearnsii<br>Spotfin Croaker<br>FMP 46	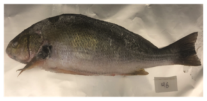 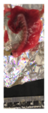
			Seriphus politus<br>Queen Croaker<br>FMP 13	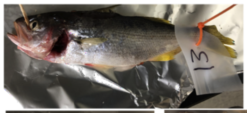 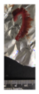
			Umbrina roncador<br>Yellowfin Drum<br>FMP 44	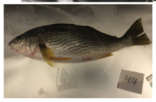 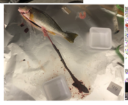 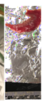
		Scorpaenidae (scorpionfish)	Scorpaena guttata<br>California Scorpionfish<br>FMP 41	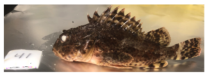 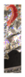
		Sebastidae (rockfish)	Sebastes auriculatus<br>Brown Rockfish<br>FMP 42	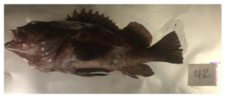 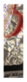
			Sebastes carnatus<br>Gopher Rockfish<br>FMP 5	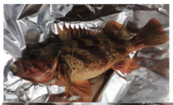 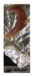
			Sebastes chlorostictus<br>Greenspotted Rockfish<br>FMP 39	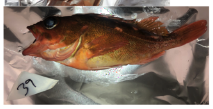 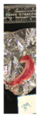
			Sebastes constellatus<br>Starry Rockfish<br>FMP 4	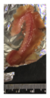
			Sebastes dallii<br>Calico Rockfish<br>FMP 27	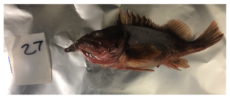 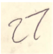 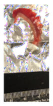

Class	Order	Family	Species/FMP number<br>Red=gill only [Atlantic]	
Actinopterygii	Perciformes	Sebastidae<br>(rockfish)	Sebastes diploproa<br>Splitnose Rockfish<br>FMP 63	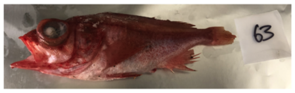 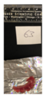
			Sebastes hopkinsi<br>Squarespot Rockfish<br>FMP 38	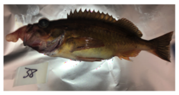 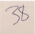 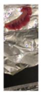
			Sebastes miniatus<br>Vermillion Rockfish<br>FMP 28	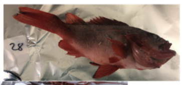 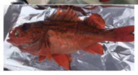 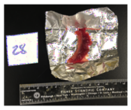
			Sebastes mystinus<br>Blue Rockfish<br>FMP 6	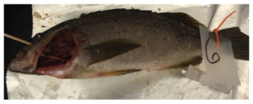 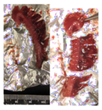
			Sebastes semicinctus<br>Halfbanded Rockfish<br>FMP 29	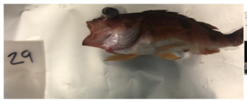 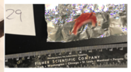
			Sebastes serriceps<br>Treefish<br>FMP 17	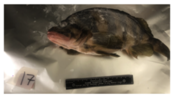 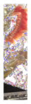
			Sebastes umbrosus<br>Honeycomb Rockfish<br>FMP 3	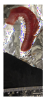
		Serranidae<br>(sea basses,<br>groupers,<br>& fairy basslets	Centropristis striata<br>Black Seabass<br>FMP 106 (A3)	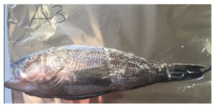
			Paralabrax clathratus<br>Kelp Bass<br>FMP 1	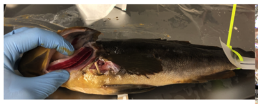 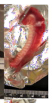
			Paralabrax maculatofasciatus<br>Spotted Sand Bass<br>FMP 35	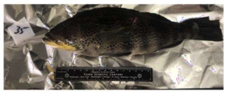 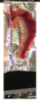
			Paralabrax nebulifer<br>Barred Sand Bass<br>FMP 12	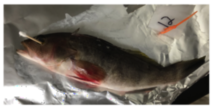 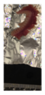
		Triglidae<br>(searobins)	Prionotus carolinus<br>Northern Searobin<br>FMP 105 (A2)	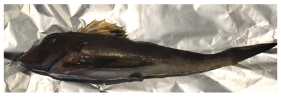

Class	Order	Family	Species/FMP number<br>Red=gill only [Atlantic]	
Actinopterygii	Perciformes	Zoarcidae<br>(eelpouts)	Lycodes cortezianus<br>Bigfin eelpout<br>FMP 75	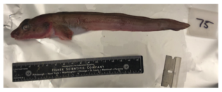 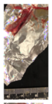
			Lycodes diapterus<br>Black Eelpout<br>FMP 82	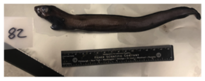 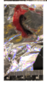
			Lycodes pacificus<br>Blackbelly Eelpout<br>FMP 85	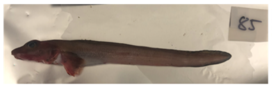 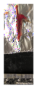
			Lyconema barbatum<br>Bearded Eelpout<br>FMP 76	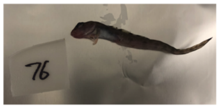 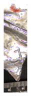
	Pleuronectiformes	Cynoglossidae<br>(tonguefish)	Symphurus atricaudus<br>California Tonguefish<br>FMP 43	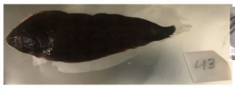 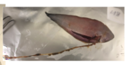 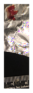
		Paralichthyidae<br>(large tooth<br>flounders)	Citharichthys sordidus<br>Pacific Sanddab<br>FMP 71	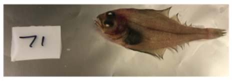 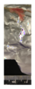
			Citharichthys xanthostigma<br>Longfin Sanddab<br>FMP 26	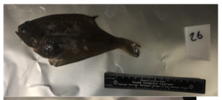 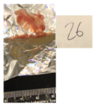
			Paralichthys californicus<br>California Flounder (Halibut)<br>FMP 37	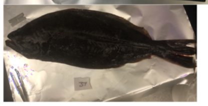 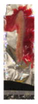
			Paralichthys dentatus<br>Summer Flounder<br>FMP 104 (A1)	
		Pleuronectidae<br>(righteye<br>flounders)	Glyptocephalus zachirus<br>Rex Sole<br>FMP 66	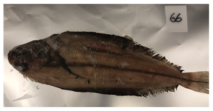 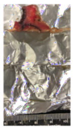
			Hypsopsetta guttulata<br>Diamond Turbot<br>FMP 101	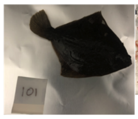 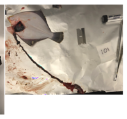
			Lyopsetta exilis<br>Slender sole<br>FMP 62	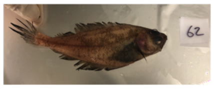 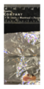
			Microstomus pacificus<br>Dover Sole<br>FMP 65	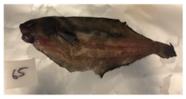 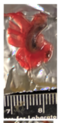
			Parophrys vetulus<br>English Sole<br>FMP 73	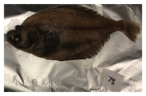 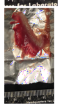

Class	Order	Family	Species/FMP number<br>Red=gill only [Atlantic]	
Actinopterygii	Scombriformes	Pomatomidae (bluefishes)	Pomatomus saltatrix<br>Bluefish<br>FMP 119 (A22)	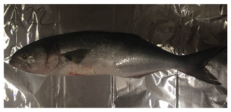
		Scombridae (mackerel, tunas, bonitos)	Katsuwonus pelamis<br>Skipjack Tuna<br>FMP 98	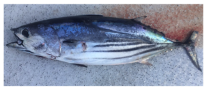
			Sarda Chiliensis<br>Pacific Bonito<br>FMP 58	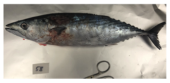 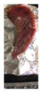
			Scomber japonicus<br>Pacific Chub Mackerel<br>FMP 103	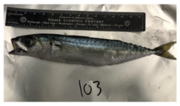 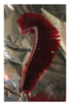
			Thunnus albacares<br>Yellowfin Tuna<br>FMP 118 (A21)	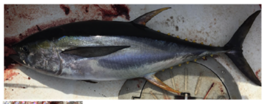
			Thunnus albacares<br>Yellowfin Tuna<br>FMP 94	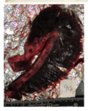
	Stomiatiformes	Sternoptychidae (marine hatchetfish)	Argyropelecus affinis<br>Pacific Hatchetfish<br>FMP 90	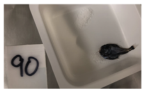 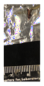
			Sternoptyx pseudobscura<br>Highlight Hatchetfish<br>FMP 89	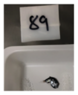 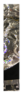
		Stomiidae (barbeled dragonfishes)	Stomias atriventer<br>Black Belly Dragonfish<br>FMP 83	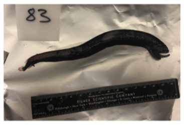 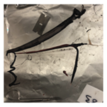 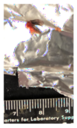
	Syngnathiformes	Syngnathidae (pipefishes & seahorses)	Syngnathus leptorhynchus<br>Bay Pipefish<br>FMP 25	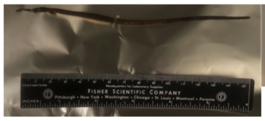 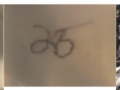 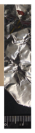

Class	Order	Family	Species/FMP number<br>Red=gill only [Atlantic]	
Chondricthyes	Carcharhiniformes	Scyliorhinidae (catsharks)	Apristurus brunneus<br>brown catshark<br>FMP 84	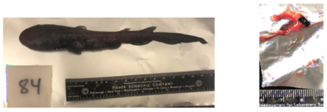
		Triakidae (houndsharks)	Mustelus californicus<br>gray smooth-hound<br>FMP 14	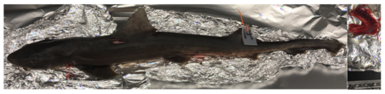
			Triakis semifasciata<br>leopard shark<br>FMP 15	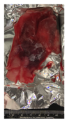
	Heterodontiformes	Heterodontidae (bullhead, horn, or port jackson sharks)	Heterodontus francisci<br>horn shark<br>FMP 32	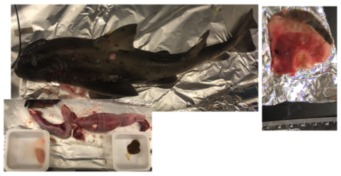
	Hexanchiformes	Hexanchidae (cow sharks)	Notorhynchus cepedianus<br>seven gill shark<br>FMP 55	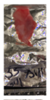
	Lamniformes	Alopiidae (thresher sharks)	Alopias vulpinus<br>thresher shark<br>FMP 59	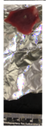
Chondrichthyes	Myliobatiformes	Dasyatidae (stingrays)	Pteroplatytrygon violacea<br>pelagic ray<br>FMP 80	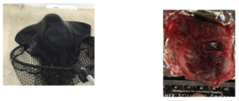
		Gymnuridae (butterfly rays)	Gymnura marmorata<br>butterfly ray<br>FMP 67	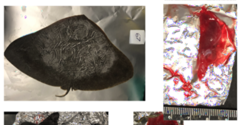
		Myliobatidae (eagle & manta rays)	Myliobatis californica<br>bat ray<br>FMP 31	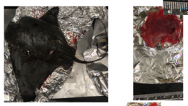
		Urotrygonidae (American round stingrays)	Urolophus halleri<br>hallers round ray<br>FMP 47	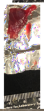
	Rajiformes	Rajidae (skates)	Leucoraja erinacea<br>little skate<br>FMP 117 (A20)	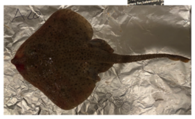
Myxini	Myxiniformes	Myxinidae (hagfishes)	Eptatretus stoutii<br>Pacific hagfish<br>FMP 81	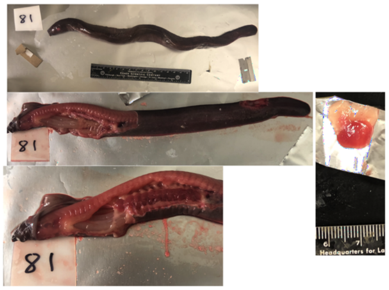

Supplementary Figure 1. Atlas of fishes from the Fish Microbiome Project 'FMP'. Samples are listed alphabetically by Class, Order, and then family. The 'FMP #' refers to the unique identifier per individual which is also in the metadata file. Species in black font are from the Pacific Ocean and have gill, skin, midgut, and hindgut microbiome samples whereas samples highlighted in red are from the Atlantic and only have gill microbiome samples. Where possible, pictures of the fish and gill sample are included.

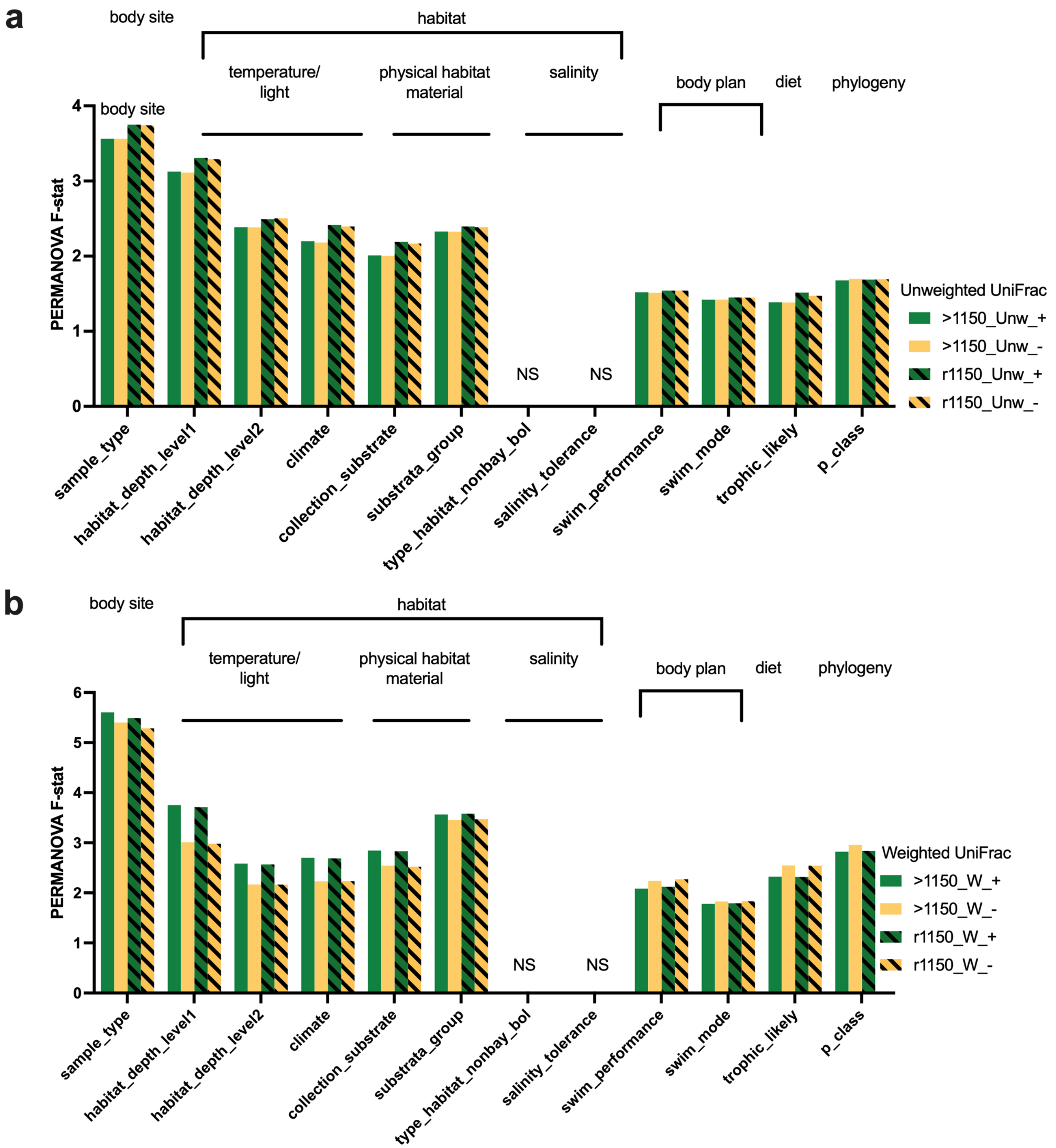

Supplementary Figure 2. Comparison of data processing methods (rarefying and removal of chloroplasts) on beta diversity significance testing using (a) Unweighted UniFrac and (b) Weighted UniFrac distances. Comparison of exclusion of samples with less than 1150 reads (non-rarified) (hashed bards) vs. rarefying at 1150 reads (clear bar). Comparison of datasets with (green bar +) and without (yellow bar -) chloroplast ASVs.

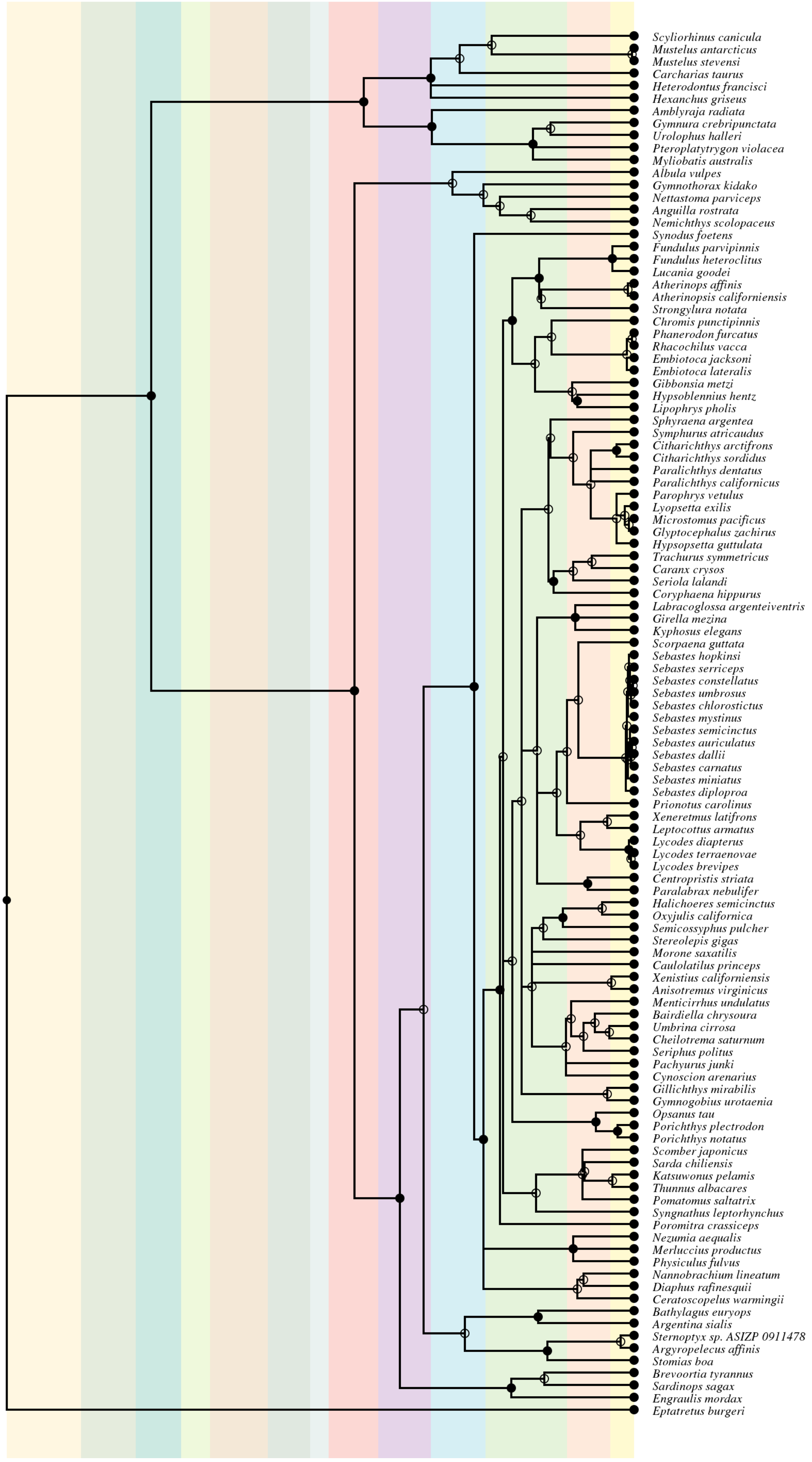

Supplementary Figure 3. Phylogenetic tree of fish species used in “FMP” beta diversity analyses

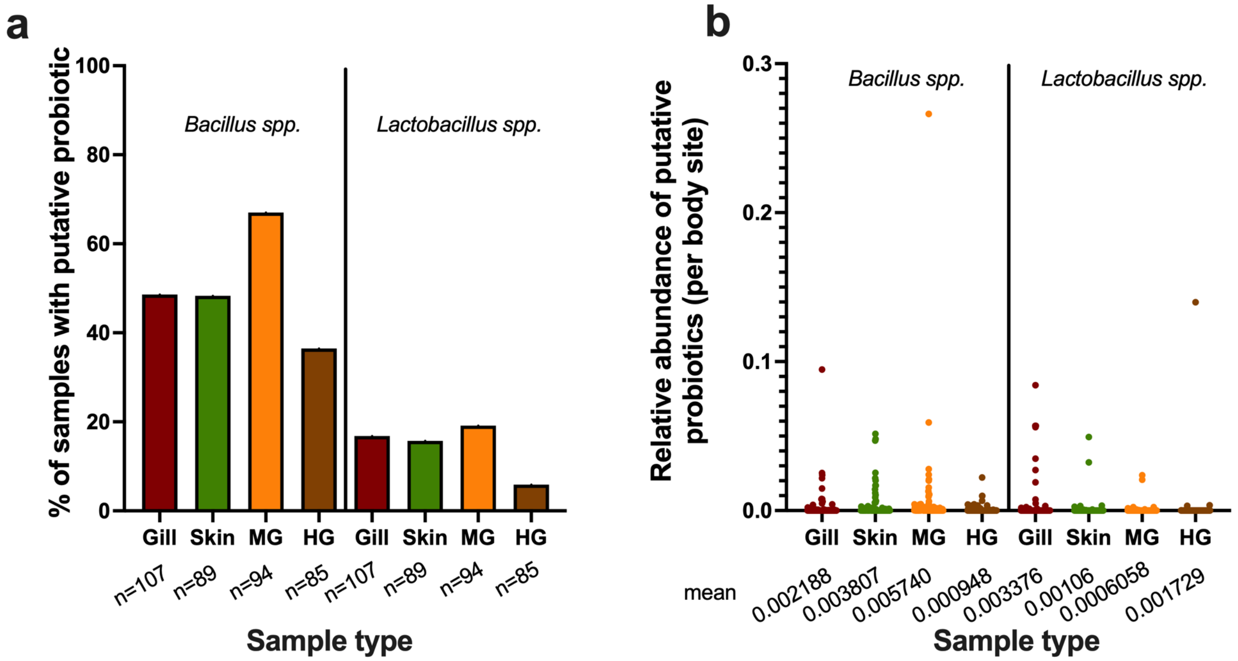

Supplementary Figure 4. Distribution of ASVs from genera with common probiotic members: Bacillus or Lactobacillus spp. a) The total percentage of samples within a given body site having either a Bacillus ASV or Lactobacillus ASV. b) The relative abundances of Bacillus and Lactobacillus ASVs within each body site (including samples with 0 counts).

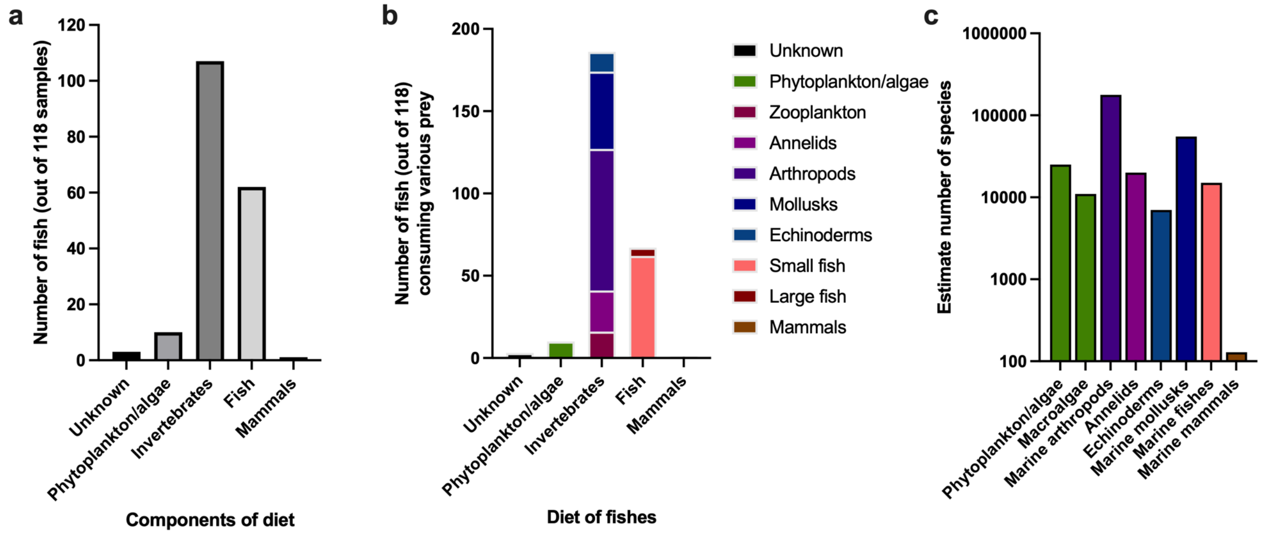

Supplementary Figure 5. General prey diversity across 118 fish (116 species) samples. Prey types of each fish determined using fishbase.org or primary literature. a) Total number of fishes (of the 118) which consume phytoplankton or algae, invertebrates, fish, or mammals. Prey data for 3 species of fish were not available (unknown). b) Higher resolution breakdown of prey consumption of fishes. c) Biodiversity estimates from the various algal, invertebrate phylums, marine fish, and marine mammal groups.

Supplementary Table 1. Statistical summary of impacts of various fish related ecological and biological factors which impact alpha diversity metrics and microbial biomass across gill, skin, midgut, and hindgut. Statistical differences determined using non-parametric testing Kruskal-Wallis test with 0.05 FDR Benjamini-Hochberg or Spearman correlation.

			Gill			Skin			Midgut			Hindgut		
**Chao1**	data type	test	P	DF	Test_stat	P	DF	Test_stat	P	DF	Test_stat	P	DF	Test_stat
habitat_depth_level1	categorical	Kruskal-Wallis	0.054	2		0.7306	2		**0.00689**	2	9.955	**0.04743**	2	6.0971
habitat_depth_level2	categorical	Kruskal-Wallis	0.0525	5		0.1308	5		**0.02228**	5	13.12	**0.01565**	4	12.241
climate	categorical	Kruskal-Wallis	0.2271	3		0.8735	3		0.06585	3		0.2381	2	
collection_substrate	categorical	Kruskal-Wallis	0.29	4		0.8401	4		**0.0343**	4	10.393	0.4206	3	
substata_group	categorical	Kruskal-Wallis	0.54	3		0.09135	3		**0.04519**	3	8.0402	0.07491	3	
type_habitat_nonbay	categorical	Kruskal-Wallis	0.3366	1		0.1799	1		0.5262	1		0.4136	1	
salinity_tolerance	categorical	Kruskal-Wallis	0.34	1		0.4911	1		0.5053	1		0.3569	1	
swim_performance	categorical	Kruskal-Wallis	0.78	5		0.4254	5		0.328	5		0.957	5	
swim_mode	categorical	Kruskal-Wallis	0.229	13		0.6693	12		0.1676	12		0.684	12	
p_class	categorical	Kruskal-Wallis	0.2186	2		0.2312	2		0.3868	2		0.4207	2	
trophic_likely	num	spearman	0.1234	NaN		0.8008	NaN		0.3814	NaN		0.1932	NaN	
ratio_gi_to_tl	num	spearman	0.8996	NaN		0.464	NaN		0.8043	NaN		0.586	NaN	
ratio_gape_to_tl	num	spearman	0.5273	NaN		0.3748	NaN		0.3484	NaN		0.1194	NaN	
swim_acceleration	int	spearman	0.5324	NaN		0.9184	NaN		0.2198	NaN		0.7862	NaN	
swim_endurance	int	spearman	0.9995	NaN		0.7875	NaN		0.3709	NaN		0.6237	NaN	
ratio_dorsal_to_tl	num	spearman	**0.02631**	NaN	-0.22558	0.08372	NaN		**0.00155**	NaN	-0.33824	0.2594	NaN	
biomass_even	num	spearman	0.6082	NaN		0.3042	NaN		**1.76E-07**	NaN	0.510102	0.1174	NaN	
biomass_quartile	num	spearman	0.2003	NaN		**0.0451**	NaN	0.2129606	**1.30E-07**	NaN	0.514797	0.1131	NaN	
ph_cells_per_g_log	num	spearman	0.2431	NaN		0.09477	NaN		**4.46E-07**	NaN	0.501626	0.1208	NaN	
**Shannon**	data type	test	P	DF	Test_stat	P	DF	Test_stat	P	DF	Test_stat	P	DF	Test_stat
habitat_depth_level1	categorical	Kruskal-Wallis	**0.03402**	2	6.7619	0.8011	2		**0.00836**	2	9.5694	0.05277	2	
habitat_depth_level2	categorical	Kruskal-Wallis	0.05729	5		0.2177	5		**0.01708**	5	13.778	0.06563	4	
climate	categorical	Kruskal-Wallis	0.1934	3		0.9315	3		0.05431	3		0.07961	2	
collection_substrate	categorical	Kruskal-Wallis	**0.03763**	4	10.172	0.8662	4		**0.04056**	4	9.9924	**0.0324**	3	8.7772
substata_group	categorical	Kruskal-Wallis	0.2936	3		0.2282	3		**0.02762**	3	9.1289	**0.00787**	3	11.862
type_habitat_nonbay	categorical	Kruskal-Wallis	0.9549	1		0.2069	1		0.9572	1		0.644	1	
salinity_tolerance	categorical	Kruskal-Wallis	0.1713	1		0.9793	1		0.3659	1		0.705	1	
swim_performance	categorical	Kruskal-Wallis	0.2697	5		0.1828	5		0.1064	5		0.2363	5	
swim_mode	categorical	Kruskal-Wallis	0.2373	13		0.224	12		0.1527	12		0.2598	12	
p_class	categorical	Kruskal-Wallis	0.1121	2		0.2813	2		0.2882	2		0.4955	2	
trophic_likely	num	spearman	0.5493	NaN		0.705	NaN		0.6288	NaN		**0.01519**	NaN	-0.26257
ratio_gi_to_tl	num	spearman	0.281	NaN		0.6274	NaN		0.6081	NaN		0.1448	NaN	
ratio_gape_to_tl	num	spearman	0.726	NaN		0.6031	NaN		**0.01915**	NaN	-0.25367	**0.0336**	NaN	-0.24249
swim_acceleration	int	spearman	0.05796	NaN		0.7916	NaN		0.09788	NaN		0.9825	NaN	
swim_endurance	int	spearman	**0.01436**	NaN	-0.23719	0.6419	NaN		0.1571	NaN		0.6202	NaN	
ratio_dorsal_to_tl	num	spearman	0.4429	NaN		0.196	NaN		**0.00832**	NaN	-0.28449	0.9591	NaN	
biomass_even	num	spearman	**1.59E-06**	NaN	-0.44666	**0.0364**	NaN	-0.222162	0.3136	NaN		**1.35E-05**	NaN	-0.45291
biomass_quartile	num	spearman	**1.40E-05**	NaN	-0.40825	0.1049	NaN		0.3996	NaN		**7.07E-06**	NaN	-0.46572
ph_cells_per_g_log	num	spearman	**4.66E-06**	NaN	-0.43224	0.07402	NaN		0.5658	NaN		**7.68E-06**	NaN	-0.4691
**Faith PD**	data type	test	P	DF	Test_stat	P	DF	Test_stat	P	DF	Test_stat	P	DF	Test_stat
habitat_depth_level1	categorical	Kruskal-Wallis	0.06199	2		0.8514	2		**0.00668**	2	10.018	0.09861	2	
habitat_depth_level2	categorical	Kruskal-Wallis	0.05567	5		0.1678	5		**0.03182**	5	12.226	**0.00715**	4	14.046
climate	categorical	Kruskal-Wallis	0.3642	3		0.9822	3		0.09907	3		0.519	2	
collection_substrate	categorical	Kruskal-Wallis	0.2859	4		0.9686	4		**0.02035**	4	11.627	0.7277	3	
substata_group	categorical	Kruskal-Wallis	0.4299	3		0.1178	3		**0.02543**	3	9.3107	0.1289	3	
type_habitat_nonbay	categorical	Kruskal-Wallis	0.3744	1		0.1524	1		0.5911	1		0.749	1	
salinity_tolerance	categorical	Kruskal-Wallis	0.5024	1		0.4206	1		0.4254	1		0.5363	1	
swim_performance	categorical	Kruskal-Wallis	0.5949	5		0.4538	5		0.3856	5		0.7706	5	
swim_mode	categorical	Kruskal-Wallis	0.3698	13		0.7793	12		0.1896	12		0.5617	12	
p_class	categorical	Kruskal-Wallis	0.4577	2		0.2977	2		0.5079	2		0.2754	2	
trophic_likely	num	spearman	0.1524	NaN		0.7742	NaN		0.4673			0.3199	NaN	
ratio_gi_to_tl	num	spearman	0.9098	NaN		0.5587	NaN		0.7104	NaN		0.9407	NaN	
ratio_gape_to_tl	num	spearman	0.7035	NaN		0.3992	NaN		0.188	NaN		0.1626	NaN	
swim_acceleration	int	spearman	0.419	NaN		0.8531	NaN		0.2194	NaN		0.7964	NaN	
swim_endurance	int	spearman	0.7854	NaN		0.7576	NaN		0.3664	NaN		0.9367	NaN	
ratio_dorsal_to_tl	num	spearman	**0.01292**	NaN	-0.2516	0.05564	NaN		**0.00079**	NaN	-0.35709	0.06643	NaN	
biomass_even	num	spearman	0.1568	NaN		0.1164	NaN		**6.04E-07**	NaN	0.490416	0.0534	NaN	
biomass_quartile	num	spearman	**0.04023**	NaN	0.199601	**0.01554**	NaN	0.2558162	**5.30E-07**	NaN	0.492548	**0.04766**	NaN	0.215463
ph_cells_per_g_log	num	spearman	**0.04152**	NaN	0.198499	**0.04426**	NaN	0.2139598	**1.45E-06**	NaN	0.481066	0.05584	NaN	
	ph_cells_per_g_log		gill			skin			mg			HG		
**Microbial biomass**	data type	test	P	DF	Test_stat	P	DF	Test_stat	P	DF	Test_stat	P	DF	Test_stat
habitat_depth_level1	categorical	Kruskal-Wallis	0.4973	2		0.3116	2		0.2363	2		0.1889	2	
habitat_depth_level2	categorical	Kruskal-Wallis	0.7477	5		0.4609	5		0.6008	5		0.3841	4	
climate	categorical	Kruskal-Wallis	0.2052	3		0.6783	3		0.1182	3		0.05414	2	5.8322
collection_substrate	categorical	Kruskal-Wallis	0.1801	4		0.1961	4		0.4204	4		0.4018	3	
substata_group	categorical	Kruskal-Wallis	**0.02291**	3	9.5403	0.1048	3		0.2051	3		0.5502	3	
type_habitat_nonbay	categorical	Kruskal-Wallis	0.6864	1		0.528	1		0.6518	1		0.7134	1	
salinity_tolerance	categorical	Kruskal-Wallis	0.6319	1		0.2584	1		0.225	1		0.109	1	
swim_performance	categorical	Kruskal-Wallis	0.2284	5		0.3843	5		0.273	5		**0.02564**	5	12.769
swim_mode	categorical	Kruskal-Wallis	**0.01312**	13	26.832	0.3909	12		0.1257	12		**0.04658**	12	21.269
p_class	categorical	Kruskal-Wallis	0.156	2		0.2543	2		0.2033	2		0.04089	2	6.3935
trophic_likely	num	spearman	0.6482	NaN		0.6224	NaN		0.1226	NaN		0.07442	NaN	
ratio_gi_to_tl	num	spearman	0.3304	NaN		0.1103	NaN		0.7613	NaN		0.6867	NaN	
ratio_gape_to_tl	num	spearman	0.8674	NaN		0.325	NaN		**0.00489**	NaN	0.302559	**0.00518**	NaN	0.315548
swim_acceleration	int	spearman	0.08945	NaN		0.5215	NaN		0.7244	NaN		0.2864	NaN	
swim_endurance	int	spearman	**0.03449**	NaN	0.205593	0.4426	NaN		0.4862	NaN		0.2167	NaN	
ratio_dorsal_to_tl	num	spearman	0.4076	NaN		0.8184	NaN		**0.03426**	NaN	-0.22993	0.3799	NaN	
biomass_even	num	Kruskal-Wallis	**2.20E-16**	NaN	0.94225	**2.20E-16**	NaN	0.9280601	**2.20E-16**	NaN	0.920848	**2.20E-16**	NaN	0.936054
biomass_quartile	num	spearman	**2.20E-16**	NaN	0.966155	**2.20E-16**	NaN	0.9561683	**2.20E-16**	NaN	0.953939	**2.20E-16**	NaN	0.967101
ph_cells_per_g_log	num	spearman	**2.20E-16**	NaN	1	**2.20E-16**	NaN	1	2.20E-16	NaN	1	**2.20E-16**	NaN	1

P value: alpha of < 0.05 is deemed significant

Test_stat: for Kruskal-Wallis this is the KW chi-squared value; for Spearman this is the rho

Supplementary Table 2. Fish Microbiome Project beta diversity comparisons (>1150 reads, chloroplasts removed) (PERMANOVA test 999 permutations)

				UniFrac			
				Unweighted		Weighted Normalized	
metadata	sample_types	n	groups	P	F-stat	P	F-stat
sample_type	all	373	4	**0.001**	3.563203	**0.001**	5.4004653
habitat_depth_level1	all	373	3	**0.001**	3.111183	**0.001**	3.0116825
habitat_depth_level2	all	373	6	**0.001**	2.382921	**0.001**	2.1677826
climate	all	373	4	**0.001**	2.180165	**0.001**	2.2305827
collection_substrate	all	373	5	**0.001**	2.003564	**0.001**	2.5456653
substrata_group	all	373	4	**0.001**	2.325092	**0.001**	3.4549728
type_habitat_nonbay_bol	all	373	2	0.007	1.465906	0.02	2.1974127
salinity_tolerance	all	373	2	0.017	1.411006	0.039	1.8031727
swim_performance	all	373	6	**0.001**	1.513274	**0.001**	2.240467
swim_mode	all	373	14	**0.001**	1.422151	**0.001**	1.8267002
trophic_likely	all	373	5	**0.001**	1.383226	**0.001**	2.547956
p_class	all	373	3	**0.001**	1.699549	**0.001**	2.9599259
biomass_even	all	373	3	**0.001**	2.957394	**0.001**	4.4198047
biomass_quartile	all	373	4	**0.001**	2.387945	**0.001**	3.1632803
habitat_depth_level1	fish gill	106	3	**0.001**	1.606352	0.019	1.8149352
habitat_depth_level2	fish gill	106	6	**0.001**	1.375987	0.089	1.2788704
climate	fish gill	106	4	0.015	1.182373	0.234	1.1509462
collection_substrate	fish gill	106	5	**0.001**	1.335963	0.012	1.6371875
substrata_group	fish gill	106	4	0.002	1.27752	**0.001**	2.1374374
type_habitat_nonbay_bol	fish gill	106	2	0.278	1.054378	0.556	0.8852508
salinity_tolerance	fish gill	106	2	0.0219	1.366908	0.079	1.6248214
swim_performance	fish gill	106	6	0.034	1.12005	0.12	1.2145679
swim_mode	fish gill	106	14	0.003	1.114537	0.092	1.1826654
trophic_likely	fish gill	106	5	0.005	1.229165	0.101	1.2844024
p_class	fish gill	106	3	0.23	1.064238	0.022	1.8143494
biomass_even	fish gill	106	3	**0.001**	1.552112	0.002	2.3423161
biomass_quartile	fish gill	106	4	0.003	1.327085	0.005	1.8112575
habitat_depth_level1	fish skin	89	3	0.078	1.146415	0.811	0.7526427
habitat_depth_level2	fish skin	89	6	0.014	1.185138	0.6049	0.9290214
climate	fish skin	89	4	0.017	1.214085	0.262	1.135714
collection_substrate	fish skin	89	5	**0.001**	1.353895	0.121	1.2677684
substrata_group	fish skin	89	4	**0.001**	1.423302	0.034	1.5432163
type_habitat_nonbay_bol	fish skin	89	2	0.143	1.128166	0.352	1.0836879
salinity_tolerance	fish skin	89	2	0.22	1.074067	0.272	1.1689699
swim_performance	fish skin	89	6	0.046	1.111547	0.124	1.2302455
swim_mode	fish skin	89	13	0.057	1.069094	0.201	1.1159289
trophic_likely	fish skin	89	5	0.125	1.087794	0.336	1.0745754
p_class	fish skin	89	3	0.005	1.326792	0.1	1.5834938
biomass_even	fish skin	89	3	0.0109	1.315375	0.028	1.7005345
biomass_quartile	fish skin	89	4	0.005	1.259727	0.132	1.2881247
habitat_depth_level1	fish midgut	93	3	**0.001**	1.98129	0.021	1.8687134
habitat_depth_level2	fish midgut	93	6	**0.001**	1.467463	0.005	1.8800109
climate	fish midgut	93	4	**0.001**	1.548837	0.047	1.5460341
collection_substrate	fish midgut	93	5	**0.001**	1.583219	0.019	1.5387735
substrata_group	fish midgut	93	4	**0.001**	1.564909	0.0129	1.6852652
type_habitat_nonbay_bol	fish midgut	93	2	0.0729	1.250755	0.002	3.2984256
salinity_tolerance	fish midgut	93	2	0.281	1.049351	0.04499	1.8537692
swim_performance	fish midgut	93	6	0.042	1.132833	0.007	1.5738001
swim_mode	fish midgut	93	13	0.003	1.148924	0.0459	1.3099478
trophic_likely	fish midgut	93	5	0.1459	1.068086	0.004	2.0891194
p_class	fish midgut	93	3	0.585	0.960102	0.3019	1.0505377
biomass_even	fish midgut	93	3	**0.001**	2.183643	0.067	1.5215333
biomass_quartile	fish midgut	93	4	**0.001**	1.710799	0.012	1.6882412
habitat_depth_level1	fish hindgut	85	3	**0.001**	1.567327	0.03799	1.4482412
habitat_depth_level2	fish hindgut	85	6	**0.001**	1.599798	0.007	1.4931924
climate	fish hindgut	85	4	0.002	1.36241	0.074	1.3300087
collection_substrate	fish hindgut	85	5	0.002	1.253162	0.005	1.6627844
substrata_group	fish hindgut	85	4	**0.001**	1.359081	**0.001**	1.9353981
type_habitat_nonbay_bol	fish hindgut	85	2	0.0589	1.171704	**0.001**	2.8530377
salinity_tolerance	fish hindgut	85	2	0.186	1.08347	0.004	2.583526
swim_performance	fish hindgut	85	6	0.0089	1.144759	0.014	1.341269
swim_mode	fish hindgut	85	13	**0.001**	1.151096	0.003	1.4326217
trophic_likely	fish hindgut	85	5	0.014	1.139078	0.0609	1.3727273
p_class	fish hindgut	85	3	0.016	1.210632	**0.001**	2.3008768
biomass_even	fish hindgut	85	3	0.005	1.251164	**0.001**	2.2757049
biomass_quartile	fish hindgut	85	4	0.041	1.120123	**0.001**	1.7728684
