# Supplementary material for: Host biology, ecology and the environment influence microbial biomass and diversity in 101 marine fish species
Source: Nat Commun. 2022 Nov 17;13:6978. doi: 10.1038/s41467-022-34557-2 (PMC9671965; doi:10.1038/s41467-022-34557-2)
Supplement: Supplementary file 3 — Description of Additional Supplementary Files [file 41467_2022_34557_MOESM3_ESM.pdf]

## Description of Additional Supplementary Files

File Name: Supplementary Dataset 1

Description: Contains the full metadata associated with each of the unique fish species in the dataset. Useful as a quick reference to look up information about fish species.

File Name: Supplementary Dataset 2

Description: Contains the full metadata associated with each of the unique fish species in the dataset along with all of the unique information associated with the microbiome sample types in the dataset (fish body sites and environment). This is the metadata used in the analyses.

File Name: Supplementary Dataset 3

Description: Contains information about each of the metadata columns (commonly referred to as the data dictionary or the metadata of the metadata).

File Name: Supplementary Dataset 4

Description: Contains the data from the gamma diversity analyses across the vertebrate species.

File Name: Supplementary Dataset 5

Description: This text file contains the output data from the SourceTracker2 analysis. The data is organized in that sink samples (fish body sites: gill, skin, midgut, hindgut) are listed as rows whereas the potential source samples are as columns. The numbers in each cell represent the estimate contribution of microbial community from a given source into a given cell. The columns labeled as ‘general summed environments’ are sums of these values organized/grouped by broad environment type (beach sand, marine sediment, sea water, and unknown). Unknown is simply 1 - (all other environments). The ratio of SW to sediment

is the SW dividied by sediment. If sediment was higher then it’s the inverse with a ‘-‘. Thus a negative value here indicates that a given sink sample has higher amounts of sediment derived microbes as compared to sea water. The remaining columns are metadata associated with the sink samples.

File Name: Supplementary Dataset 6

Description: This is a text file of the annotated biom table from the Fish Microbiome Project dataset (used for analyses). This text file contains 373 samples (fish body sites: gill, skin, midgut, hindgut) and a total of 53904 features.
