# Supplementary material for: Host biology, ecology and the environment influence microbial biomass and diversity in 101 marine fish species
Source: Nat Commun. 2022 Nov 17;13:6978. doi: 10.1038/s41467-022-34557-2 (PMC9671965; doi:10.1038/s41467-022-34557-2)
Supplement: Supplementary file 10 — Reporting Summary [file 41467_2022_34557_MOESM10_ESM.pdf]

nature portfolio

Corresponding author(s): Jeremiah J Minich
Last updated by author(s): Sep 28, 2022

Reporting Summary

Nature Portfolio wishes to improve the reproducibility of the work that we publish. This form provides structure for consistency and transparency in reporting. For further information on Nature Portfolio policies, see our Editorial Policies and the Editorial Policy Checklist.

Statistics

For all statistical analyses, confirm that the following items are present in the figure legend, table legend, main text, or Methods section.

n/a	Confirmed	
☐	☒	The exact sample size (*n*) for each experimental group/condition, given as a discrete number and unit of measurement
☐	☒	A statement on whether measurements were taken from distinct samples or whether the same sample was measured repeatedly
☐	☒	The statistical test(s) used AND whether they are one- or two-sided<br>*Only common tests should be described solely by name; describe more complex techniques in the Methods section.*
☐	☒	A description of all covariates tested
☐	☒	A description of any assumptions or corrections, such as tests of normality and adjustment for multiple comparisons
☐	☒	A full description of the statistical parameters including central tendency (e.g. means) or other basic estimates (e.g. regression coefficient) AND variation (e.g. standard deviation) or associated estimates of uncertainty (e.g. confidence intervals)
☐	☒	For null hypothesis testing, the test statistic (e.g. *F*, *t*, *r*) with confidence intervals, effect sizes, degrees of freedom and *P* value noted<br>*Give P values as exact values whenever suitable.*
☒	☐	For Bayesian analysis, information on the choice of priors and Markov chain Monte Carlo settings
☒	☐	For hierarchical and complex designs, identification of the appropriate level for tests and full reporting of outcomes
☐	☒	Estimates of effect sizes (e.g. Cohen's *d*, Pearson's *r*), indicating how they were calculated

Our web collection on statistics for biologists contains articles on many of the points above.

Software and code

Policy information about availability of computer code

Data collection	microbiome software used for data processing and collection: Qiita 2022.07, Qiime2-2022.2, Deblur v1.1.0
Data analysis	microbiome software used for data analysis: Qiita 2022.07, Qiime2-2022.2, Deblur v1.1.0, Prism 9.4.1; Qiita analysis ID 49571, github.com/jminich444/Fish_Microbiome_Project;

For manuscripts utilizing custom algorithms or software that are central to the research but not yet described in published literature, software must be made available to editors and reviewers. We strongly encourage code deposition in a community repository (e.g. GitHub). See the Nature Portfolio guidelines for submitting code & software for further information.

Data

Policy information about availability of data

All manuscripts must include a data availability statement. This statement should provide the following information, where applicable:

- Accession codes, unique identifiers, or web links for publicly available datasets
- A description of any restrictions on data availability
- For clinical datasets or third party data, please ensure that the statement adheres to our policy

This statement is in the manuscript. Also it is here EBI ERP139587; Qiita study ID 13414; fishbase.org used to gather life history information on fishes

Human research participants

Policy information about studies involving human research participants and Sex and Gender in Research.

Reporting on sex and gender	N/A
Population characteristics	N/A
Recruitment	N/A
Ethics oversight	N/A

Note that full information on the approval of the study protocol must also be provided in the manuscript.

Field-specific reporting

Please select the one below that is the best fit for your research. If you are not sure, read the appropriate sections before making your selection.

☐ Life sciences ☐ Behavioural & social sciences ☒ Ecological, evolutionary & environmental sciences

For a reference copy of the document with all sections, see nature.com/documents/nr-reporting-summary-flat.pdf

Ecological, evolutionary & environmental sciences study design

All studies must disclose on these points even when the disclosure is negative.

Study description

This was a microbiome study (16S) of four body sites from 101 different species of wild marine fish. There were also additional species from the Atlantic.

Research sample

All details are in the supplemental data set "fmp_metadata": Paralabrax clathratus
Caulolatilus princeps
Sebastes umbrosus
Sebastes constellatus
Sebastes carnatus
Sebastes mystinus
Phanerodon furcatus
Halichoeres californicus
Embiotoca jacksoni
Anisotremus davidsonii
Sphyraena argentea
Paralabrax nebulifer
Seriphus politus
Mustelus californicus
Triakis semifasciata
Leptocottus armatus
Sebastes serriceps
Semicossyphus pulcher
Atherinopsis californiensis
Trachurus symmetricus
Kyphosus azureus
Girella nigricans
Heterostichus rostratus
Oxyjulis californica
Syngnathus leptorhynchus
Citharichthys xanthostigma
Sebastes dallii
Sebastes miniatus
Sebastes semicinctus
Menticirrhus undulatus
Myliobatis californica
Heterodontus francisci
Gibbonsia elegans
Hypsoblennius gilberti
Paralabrax maculatofasciatus
Chromis punctipinnis

Paralichthys californicus
Sebastes hopkinsi
Sebastes chlorostictus
Engraulis mordax
Scorpaena guttata
Sebastes auriculatus
Symphurus atricaudus
Umbrina roncador
Brachygenys californiensis
Roncador stearnsii
Urobatis halleri
Porichthys myriaster
Cynoscion parvipinnis
Albula gilberti
Synodus lucioceps
Rhacochilus toxotes
Brachyistius frenatus
Medialuna californiensis
Notorynchus cepedianus
Sarda chiliensis
Alopias vulpinus
Sardinops sagax
Triphoturus mexicanus
Lyopsetta exilis
Sebastes diploproa
Physiculus rastrelliger
Microstomus pacificus
Glyptocephalus zachirus
Gymnura marmorata
Gymnothorax mordax
Xeneretmus latifrons
Lipolagus ochotensis
Citharichthys sordidus
Porichthys notatus
Parophrys vetulus
Merluccius productus
Lycodes cortezianus
Lyconema barbatum
Leuroglossus stilbius
Nemichthys scolopaceus
Seriola dorsalis
Pteroplatytrygon violacea
Eptatretus stoutii
Lycodes diapterus
Stomias atriventer
Apristurus brunneus
Lycodes pacificus
Facciolella equatorialis
Scopelogadus (mizolepsis) bispinosus
not applicablenobrachium ritteri
Sternoptyx pseudobscura
Argyropelecus affinis
Ceratoscopelus townsendi
Stereolepis gigas
Nezumia stelgidolepis
Thunnus albacares
Clevelandia ios
Fundulus parvipinnis
Coryphaena hippurus
Katsuwonus pelamis
Atherinops affinis
Gillichthys mirabilis
Pleuronichthys guttulatus
Cheilotrema saturnum
Scomber japonicus
Paralichthys dentatus
Prionotus carolinus
Centropristis striata

Bairdiella chrysoura
Fundulus heteroclitus
Brevoortia tyrannus
Chasmodes bosquianus
Strongylura marina
Opsanus tau
Anguilla rostrata
Caranx crysos
Coryphaena hippurus
Lucania parva
Leucoraja erinacea
Thunnus albacares
Pomatomus saltatrix
Morone saxatilis

Sampling strategy	Wild fish were collected using the scientific research permit of the corresponding author. Also bycatch from other scientific collections were included. Lastly, some freshly dead fish were donated by recreational fishers. 1 fish per species was collected.
Data collection	Samples were collected and data on species type, location, time recorded immediately. Other data on mass, length, gape size, etc. were collected during the dissections. Data was collected by pen and paper and then transfered to google sheets.
Timing and spatial scale	Fish were collection opportunistically across a few years from 2018-2020. Most fish were from Southern California (San Diego area). Various groups or cohorts of fish were collected during targeted surveys such as the bottom trawl surveys conduced on the SIO research vessels.
Data exclusions	We use a very specific sample exclusion methodology based on the read counts for each sample compared to known positive controls. This is detailed in the paper. In fact, this exclusion criteria methodology is one of the primary methods developed in this paper. See the methods and qiime2 plugin katharoseq for more detail.
Reproducibility	All data is available and we have done our best to make it all public. In this instance we were collecting wild animals. One will never be able to go back in time to collect those exact animals from the exact locations so in some regards one could consider this a natural observation type of study. One could however collect the same species at a later time point to try and reproduce the findings.
Randomization	For sample processing, all species were randomized on each extraction plate but were processed generally by sample type
Blinding	Is it not possible to do blind analyses as we were looking at how different factors influence the fish microbiome community

Did the study involve field work? ☒ Yes ☐ No

Field work, collection and transport

Field conditions

Samples were collected from either small craft, research vessels, fishing from a fixed object (pier), or from spearfishing/kayaking. All sampling were conducted in safe conditions. No samples were collected during the rain. We do not have air temperatures for when samples were collected but generally speaking it would have been between 50-80 F.

Location

All exact lat/long details and depth etc are in the metadata files. Samples were collected from the EEZ of San Diego, CA or from NY New York.
hhost_scientific_name depth_m latitude longitude
Paralabrax clathratus 3 32.848152 -117.278413
Caulolatilus princeps 10 32.854483 -117.273919
Sebastes umbrosus 30 32.83399 -117.30876
Sebastes constellatus 30 32.83399 -117.30876
Sebastes carnatus 30 32.83399 -117.30876
Sebastes mystinus 30 32.83399 -117.30876
Phanerodon furcatus 3 33.113536 -117.325634
Halichoeres californicus 3 33.113536 -117.325634
Embiotoca jacksoni 3 33.113536 -117.325634
Anisotremus davidsonii 3 33.113536 -117.325634
Sphyraena argentea 2 32.80081 -117.269514
Paralabrax nebulifer 2 32.80081 -117.269514
Seriphus politus 2 32.80081 -117.269514
Mustelus californicus 3 32.788406 -117.246274
Triakis semifasciata 3 32.770985 -117.242299
Leptocottus armatus 1 32.841184 -117.281972
Sebastes serriceps 30 32.83399 -117.30876
Semicossyphus pulcher 30 32.83399 -117.30876
Atherinopsis californiensis 5 32.858496 -117.276143

Trachurus symmetricus 5 32.858496 -117.276143
Kyphosus azureus 3 32.832233 -117.28381
Girella nigricans 3 32.832233 -117.28381
Heterostichus rostratus 3 32.832233 -117.28381
Oxyjulis californica 3 32.832233 -117.28381
Syngnathus leptorhynchus 1 32.867124 -117.257229
Citharichthys xanthostigma 35 32.961121 -117.302943
Sebastes dallii 35 32.961121 -117.302943
Sebastes miniatus 35 32.961121 -117.302943
Sebastes semicinctus 35 32.961121 -117.302943
Menticirrhus undulatus 1 33.113536 -117.325634
Myliobatis californica 1 33.113536 -117.325634
Heterodontus francisci 4 32.75757 -117.249448
Gibbonsia elegans 1 32.841184 -117.281972
Hypsoblennius gilberti 1 32.841184 -117.281972
Paralabrax maculatofasciatus 3 32.760464 -117.245347
Chromis punctipinnis 3 32.760464 -117.245347
Paralichthys californicus 3 32.776937 -117.243164
Sebastes hopkinsi 50 32.709444 -117.3125
Sebastes chlorostictus 50 32.708889 -117.299722
Engraulis mordax 5 32.762464 -117.239009
Scorpaena guttata 30 33.142589 -117.378686
Sebastes auriculatus 30 33.126629 -117.374183
Symphurus atricaudus 3 32.789026 -117.24557
Umbrina roncador 3 32.772748 -117.229627
Brachygenys californiensis 3 32.772748 -117.229627
Roncador stearnsii 3 32.772748 -117.229627
Urobatis halleri 3 32.789805 -117.248309
Porichthys myriaster 3 32.776937 -117.243164
Cynoscion parvipinnis 3 32.658399 -117.232094
Albula gilberti 3 32.658399 -117.232094
Synodus lucioceps 3 32.769349 -117.227735
Rhacochilus toxotes 6 32.830845 -117.294357
Brachyistius frenatus 6 32.830845 -117.294357
Medialuna californiensis 6 32.830845 -117.294357
Notorynchus cepedianus 6 32.841484 -117.297446
Sarda chiliensis 3 32.867124 -117.257229
Alopias vulpinus 3 32.789805 -117.248309
Sardinops sagax 3 32.867124 -117.257229
Triphoturus mexicanus 300 32.674701 -117.355389
Lyopsetta exilis 300 32.674701 -117.355389
Sebastes diploproa 300 32.674701 -117.355389
Physiculus rastrelliger 300 32.674701 -117.355389
Microstomus pacificus 300 32.674701 -117.355389
Glyptocephalus zachirus 300 32.674701 -117.355389
Gymnura marmorata 1 32.769915 -117.248336
Gymnothorax mordax 1 32.763155 -117.241393
Xeneretmus latifrons 150 32.63861111 -117.3416667
Lipolagus ochotensis 500 32.70094444 -117.5519444
Citharichthys sordidus 150 32.63861111 -117.3416667
Porichthys notatus 150 32.63861111 -117.3416667
Parophrys vetulus 150 32.63861111 -117.3416667
Merluccius productus 328.5 32.68916667 -117.3844444
Lycodes cortezianus 328.5 32.68916667 -117.3844444
Lyconema barbatum 328.5 32.68916667 -117.3844444
Leuroglossus stilbius 328.5 32.68916667 -117.3844444
Nemichthys scolopaceus 500 32.70094444 -117.5519444
Seriola dorsalis 10 33.098027 -117.374053
Pteroplatytrygon violacea 3 33.098027 -117.374053
Eptatretus stoutii 300 32.674701 -117.355389
Lycodes diapterus 300 32.674701 -117.355389
Stomias atriventer 500 32.674701 -117.355389
Apristurus brunneus 500 32.674701 -117.355389
Lycodes pacificus 500 32.674701 -117.355389
Facciolella equatorialis 500 32.674701 -117.355389
Scopelogadus (mizolepsis) bispinosus 500 32.674701 -117.355389
not applicablenobrachium ritteri 500 32.674701 -117.355389
Sternoptyx pseudobscura 500 32.70094444 -117.5519444

	Argyropelecus affinis 500 32.70094444 -117.5519444<br>Ceratoscopelus townsendi 500 32.70094444 -117.5519444<br>Stereolepis gigas 10 32.861218 -117.274588<br>Nezumia stelgidolepis 500 32.674701 -117.355389<br>Thunnus albacares 1 32.091667 -118.208333<br>Clevelandia ios 1 32.769594 -117.248478<br>Fundulus parvipinnis 1 32.782075 -117.250127<br>Coryphaena hippurus 3 32.616069 -117.396926<br>Katsuwonus pelamis 3 31.435833 -117.420556<br>Atherinops affinis 1 32.787057 -117.208794<br>Gillichthys mirabilis 1 32.787057 -117.208794<br>Pleuronichthys guttulatus 1 32.769594 -117.248478<br>Cheilotrema saturnum 2 32.748242 -117.255313<br>Scomber japonicus 1 32.867124 -117.257229<br>Paralichthys dentatus 30 40.545278 -73.046028<br>Prionotus carolinus 24 40.559667 -73.037444<br>Centropristis striata 30 40.546278 -73.045222<br>Bairdiella chrysoura 1 40.664722 -73.383187<br>Fundulus heteroclitus 0.5 40.664722 -73.383187<br>Brevoortia tyrannus 2 40.664722 -73.383187<br>Chasmodes bosquianus 1 40.664722 -73.383187<br>Strongylura marina 2 40.664722 -73.383187<br>Opsanus tau 1 40.664722 -73.383187<br>Anguilla rostrata 1 40.664722 -73.383187<br>Caranx crysos 38 40.250278 -73.164167<br>Coryphaena hippurus 38 40.265722 -73.223<br>Lucania parva 0.5 40.664722 -73.383187<br>Leucoraja erinacea 22 40.539722 -73.041111<br>Thunnus albacares 152 39.524611 -72.207806<br>Pomatomus saltatrix 17.2 40.628217 -73.29205<br>Morone saxatilis 3 40.664722 -73.383187
Access & import/export	All samples were collected under permit or donated from fishers from US locations
Disturbance	No impact

Reporting for specific materials, systems and methods

We require information from authors about some types of materials, experimental systems and methods used in many studies. Here, indicate whether each material, system or method listed is relevant to your study. If you are not sure if a list item applies to your research, read the appropriate section before selecting a response.

Materials & experimental systems

n/a	Involved in the study
☒	☐ Antibodies
☒	☐ Eukaryotic cell lines
☒	☐ Palaeontology and archaeology
☐	☒ Animals and other organisms
☒	☐ Clinical data
☒	☐ Dual use research of concern

Methods

n/a	Involved in the study
☒	☐ ChIP-seq
☒	☐ Flow cytometry
☒	☐ MRI-based neuroimaging

Animals and other research organisms

Policy information about studies involving animals; ARRIVE guidelines recommended for reporting animal research, and Sex and Gender in Research

Laboratory animals	No lab animals were used in the study
Wild animals	Wild fish were caught using a variety of methods including trawl, hook and line, spear, and gillnet. They were immediately euthanized using the approved procedure of ikejime. Fishes which were caught from bycatch (moribund) were also euthanized this way if they were not already deceasesd. Most fishes one cannot know the age although we can say that most if not all of our fish were in the juvenile to adult range but not larval.

Reporting on sex	For the most part, we did not identify sex. Where we did it is in the metadata.
Field-collected samples	all samples were from the field (wild)
Ethics oversight	All animals were handled and euthanized ethically according to the universities guidelines. For collection, we used a 2016 scientific collecting permit DFW 1379: DocID: D-0018712881-8

Note that full information on the approval of the study protocol must also be provided in the manuscript.
